# The effects of metformin on anti-Müllerian hormone levels in patients with polycystic ovary syndrome: a systematic review and meta-analysis

**DOI:** 10.1186/s13048-023-01195-1

**Published:** 2023-06-28

**Authors:** Zhijiao Zhou, Hongzhi Chen, Ling Chu, Qiong Zou, Qian Chen, Jun Yang, Yang Liu, Xiang Ou

**Affiliations:** 1 Department of Pathology, Third Xiangya Hospital, Central South University, Changsha, 410013 China; 2 Key Laboratory of Diabetes Immunology, Ministry of Education, National Clinical Research Center for Metabolic Disease; Department of Metabolism and Endocrinology, Second Xiangya Hospital, Central South University, Changsha, 410013 China; 3 Department of Endocrinology, The Affiliated Changsha Central Hospital, Hengyang Medical School, University of South China, Changsha, 410004 Hunan China

**Keywords:** PCOS, AMH, Metformin, Meta-analysis

## Abstract

**Objective:**

To analyze whether metformin treatment in patients with polycystic ovary syndrome (PCOS) results in a decrease of anti-Müllerian hormone (AMH) levels, we reviewed and analyzed PCOS studies which evaluated serum AMH levels before and after metformin treatment.

**Methods:**

This is a systematic review and meta-analysis of self-controlled clinical trials. Databases including PubMed, Embase, and Web of Science library were searched to identify eligible studies published before February 2023. Random-effects models were applied to assess standardized mean differences (SMDs) with 95% confidence intervals (95% CI).

**Results:**

The electronic-based search retrieved 167 articles of which 14 studies (12 publications) involving 257 women with PCOS were included. In general, AMH levels decreased significantly after metformin treatment [SMD (95% CI) of -0.70 (-1.13 to -0.28); *P* = 0.001]. Metformin exhibited a strong inhibitory effect on AMH levels for PCOS patients with age less than 28 [SMD − 1.24, 95% CI − 2.15 to − 0.32, *P* = 0.008]. Additionally, AMH levels significantly slid down in PCOS patients with no more than 6 months metformin treatment [SMD − 1.38, 95% CI − 2.18 to − 0.58, *P* = 0.0007], or with no more than a dose of 2000 mg/day [SMD -0.70, 95% CI -1.11 to -0.28; *P* = 0.001]. Notably, suppressive effects of metformin treatment were merely observed in patients with AMH levels at baseline higher than 4.7 ng/ml [SMD − 0.66, 95% CI − 1.02 to − 0.31, *P* = 0.0003].

**Conclusion:**

This meta-analysis provided quantitative evidence demonstrating that metformin significantly decreased AMH levels, especially for young patients and those with AMH levels at baseline higher than 4.7 ng/ml.

**Trial registration:**

PROSPERO CRD42020149182.

**Supplementary Information:**

The online version contains supplementary material available at 10.1186/s13048-023-01195-1.

## Introduction

Affecting 6%–10% women of reproductive age, polycystic ovary syndrome (PCOS) is one of the most common endocrine disorders [1]. Produced in granulosa cells, anti-Müllerian hormone (AMH) serves as an indicator of ovarian reserve based on its ability to reflect the number of antral and pre-antral follicles [2]. While AMH is essential in folliculogenesis, excessive AMH also contributes to anovulation by counteracting FSH in follicle growth and maturation [3]. A correlation of serum AMH levels with different PCOS phenotypes was observed [4]. Consequently, AMH measurement is increasingly considered a surrogate for polycystic ovarian morphology [5] as the patients present elevated serum AMH levels which are 2 ~ 3 folders higher [6].

Metformin is often prescribed to PCOS patients. Besides alleviating insulin resistance of PCOS patients, metformin is also effective in menstrual cycle restoration and ovulation induction [7–9]. As of yet it remains not fully understood how metformin improves reproductive function of PCOS patients. A number of studies were carried out to determine whether metformin treatment could regulate AMH levels in PCOS patients. Some of the trials depicted a decrease of AMH levels after metformin treatment [6, 10, 11], but some reported no changes of AMH levels [12, 13]. It is vital to determine whether metformin affect the levels of serum AMH in women with PCOS or not. It is also critical to uncover the clinical characteristics of the enrolled PCOS patients in the trials. Therefore, we reviewed the relevant literature systematically and performed meta-analysis on the data collected to determine the effect of metformin treatment on AMH levels in PCOS patients.

## Methods

This study was performed in accordance with the Preferred Reporting Items for Systematic Reviews and Meta-analyses (PRISMA) statement (Supplementary Table 1) [14].

### Search strategy

In this study, two investigators (ZZ, CH) independently searched the databases of PubMed, Embase, and Web of Science for original articles from database inception to October 1, 2019. The search strategy of the current study was limited to full text articles published in English. The search terms used were: (anti-Müllerian hormone or AMH or Mullerian inhibiting substance *o*r MIS) AND (metformin) AND (PCOS *or* polycystic ovary syndrome). A search update was conducted on February 5, 2023. Besides, the investigators manually screened the reference lists of included studies to identify any relevant publications missed in the electronic search.

### Literature selection criteria

Articles meeting all the following criteria were included: (1) women with PCOS receiving metformin treatment in self-controlled clinical trials (2); AMH means and standard deviations (SDs) reported or enough information provided so that AMH means and SDs could be calculated (3); the study employed Rotterdam criteria, National Institutes of Health (NIH) criteria, or Androgen Excess and Polycystic Ovarian Syndrome Society 2006 (AE/PCOS) criteria for PCOS diagnosis; (4) published in English and in full text format. Articles were excluded if they were published as abstracts, letters, reviews, expert comments, editors’ opinions, animal studies, or case reports.

### Data extraction

All information from each eligible article was extracted by two reviewers (ZZ, CH) independently using one standardized data extraction form. The extracted information included: study design, first author, year of publication, age of women with PCOS, number of women with PCOS, dose and duration of metformin, levels and measurement methods of AMH. Only data from the most recent point in time were extracted if duplicate data were found. Disagreements upon information obtained by different reviewers were settled through discussion. Furthermore, we contacted the primary authors to acquire and verify the information when necessary.

### Statistical analysis

The primary outcome was the alterations of AMH levels in women with PCOS before and after metformin treatment. We assessed the methodological quality of the included studies according to the Newcastle–Ottawa scale (NOS). NOS fulfills the following three domains: selection, comparability, and outcome. The studies were allocated stars which ranging from zero to nine according to the risk of bias (Supplementary Table 2).

The standard mean difference (SMD) and 95% confidence interval (CI) for AMH levels were calculated using the fixed-effect model when there was no or low statistical heterogeneity (*I*^2^ < 50%). The random-effect model was chosen when there was moderate or high heterogeneity (*I*^2^ ≥ 50%). To determine the sources of heterogeneity, we performed subgroup analyses which included age, duration and dose of treatment, and AMH levels at baseline. In addition, sensitivity analysis was conducted to evaluate the stability of the meta-analysis result. Potential publication bias was studied via the funnel plot, the Begg’s test and the Egger’s test. Statistical analysis was performed using Review Manager (Revman 5.3; Cochrane Collaboration, Copenhagen, Denmark) and STATA 12.0 (Stata Corp. College Station, TX, USA), *P* < 0.05 was considered statistically significant.

## Results

### Study selection

**Fig. 1:**
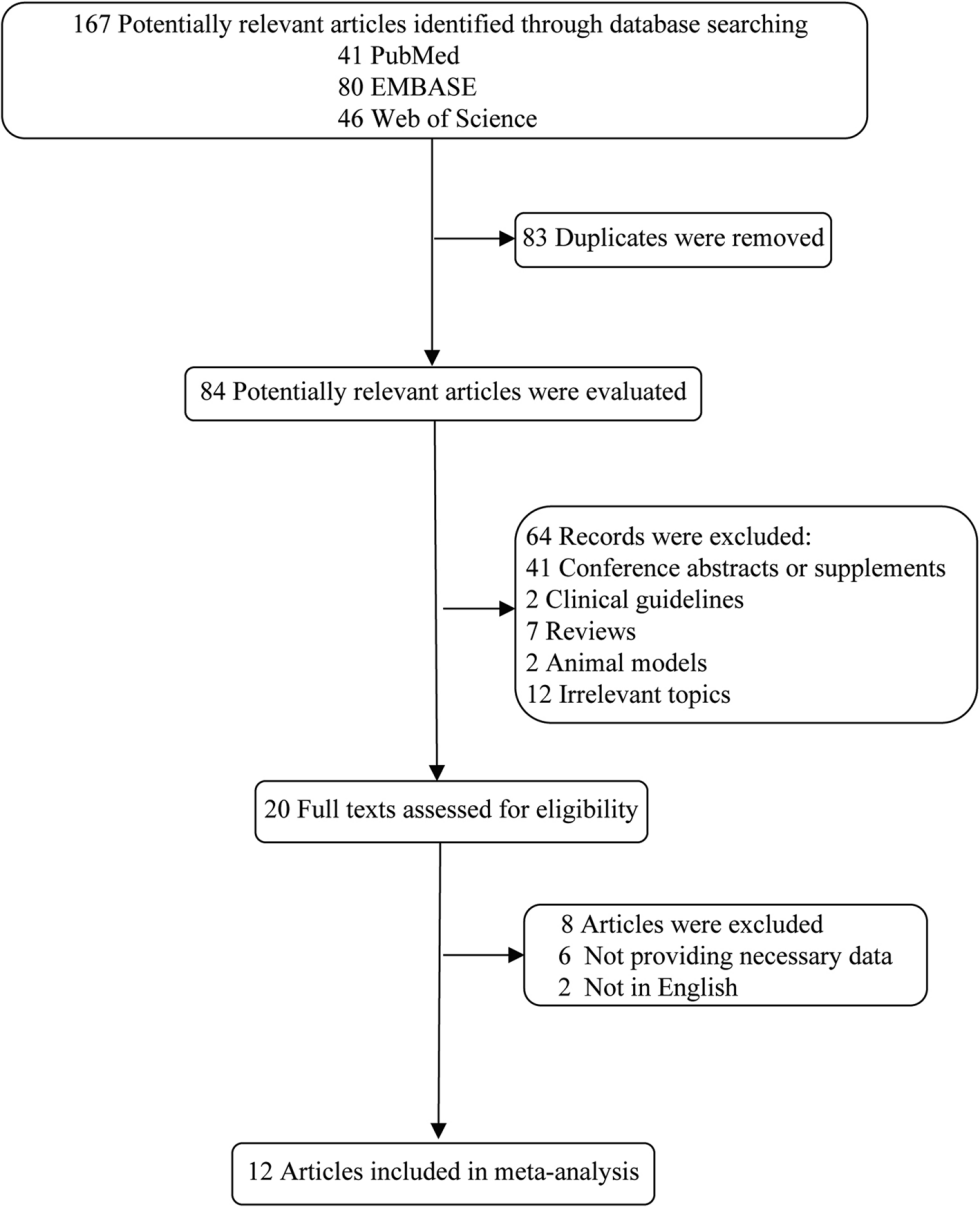
Search flow diagram

A flow chart of relevant research articles filtration is shown in Fig. 1. Based on the search strategy, 167 articles were identified from electronic databases of PubMed, Embase, and Web of Science. After excluding the duplicates (*n* = 83), reviewers selected 84 papers after screening the titles and abstracts. Among these 84 articles, 64 were excluded for the following reasons: 41 conference abstracts and supplements, 2 clinical guidelines, 7 reviews, 2 animal studies, and 12 irrelevant studies. Therefore, 20 full articles were assessed for eligibility. Among them, we further excluded 8 for the following reasons: 6 failed to gain access to the necessary information, 2 were not in English. Finally, 12 articles were included in the meta-analysis.

### Study characteristics

Twelve articles representing 257 PCOS patients were considered appropriate for the meta-analysis (Table 1). The studies by Grigoryan et al. [15] and Tomova et al. [16] were each divided into two following the grouping ways the original articles indicated. Therefore, the meta-analysis included a total of 14 studies. The NIH criteria were employed in the studies carried by Panidis [12] and Bayrak [17]; The study conducted by Neagu [18] employed the AE/PCOS diagnostic criteria; The Rotterdam criteria were employed in other included studies [6, 10, 11, 13, 15, 16, 19–21].

**Table 1:** Characteristics of the 14 studies in the meta-analysis

First Author	Year	Country	PCOS criteria	Age (Mean±SDPre/Post)	Number (Pre/Post)	BMI (Mean±SDPre/Post)	Study design	Dose(mg/ day)	Therapymonth	Method	AMH (mean±SD)	
											Pre	Post
Piltonen	2005	Finland	Rotterdam	21-41	26	18-41	SCT	mean 1500	6	EIA	12.25±2.1	11.4±2.24
Bayrak	2007	USA	NIH	29.5±4.8	10	30.2±4.5	SCT	850	1/4	ELISA	2.82±1.8	2.92±1.6
Carlsen	2009	Norway	Rotterdam	30.6±5.9	20	33.4±7.5	SCT	2550	6	ELISA	15.3±11.5	15.2±12.0
Tomova-a	2011	Bulgaria	Rotterdam	NA	13	33.8±2.76 Vs	SCT	2550	6	ELISA	6.39±1.3	5.36±0.96
						31.35±2.80						
Tomova-b	2011	Bulgaria	Rotterdam	NA	4	22.08±1.12 Vs	SCT	2550	6	ELISA	4.38±2.31	11.31±1.78
						22.08±1.32						
Panidis	2011	Greece	NIH	20.53±3.09	15	21.97±1.69 vs	SCT	1700	6	ELISA	9.24±3.70	7.77±2.82
						20.75±1.32						
Neagu	2012	Romania	AE/PCOS	NA	11	NA	SCT	gradually increase to 2550	2	NA	8.99±0.99	6.28.±0.46
Nascimento	2013	Brazil	Rotterdam	26.3±1	16	29.1±1.7	SCT	1500	2	ELISA	6.99±0.85	5.81±0.78
Grigoryan-a	2014	Russia	Rotterdam	28.7±5.2	18	NA	SCT	1700	6	EIA	8.1±1.8	6.9±1.6
Grigoryan-b	2014	Russia	Rotterdam	28.7±5.2	22	NA	SCT	1700	6	EIA	7.7±1.4	7.9±1.6
Saleh	2015	Iraq	Rotterdam	27.5±1.32	20	30.85±0.95 Vs	SCT	1500	3	ELISA	4.49±0.54	3.03±0.53
						29.01±1.01						
Foroozanfard	2017	Iran	Rotterdam	25.2±4.2	30	26.2±3.8	SCT	gradually increase to 1500	2	ELISA	10±3.75	7.8±3.7
Wiweko	2017	Indonesia	Rotterdam	28.25±3.99	20	28.02±6.02Vs	SCT	1500	6	Beckman	9.30±5.06	7.47±4.59
						27.6±6.13				Coulter gen II		
										AMH assay		
Chhabra	2018	India	Rotterdam	30.03±3	32	NA	SCT	1700	3	NA	11.87±5.6	7.4±3.9

*NA* not available, *NIH* National Institutes of Health, *SCT* self-control trial, *SD* standard deviation, *Pre* before metformin, *Post* after metformin

### Synthesis of results

**Fig. 2:**
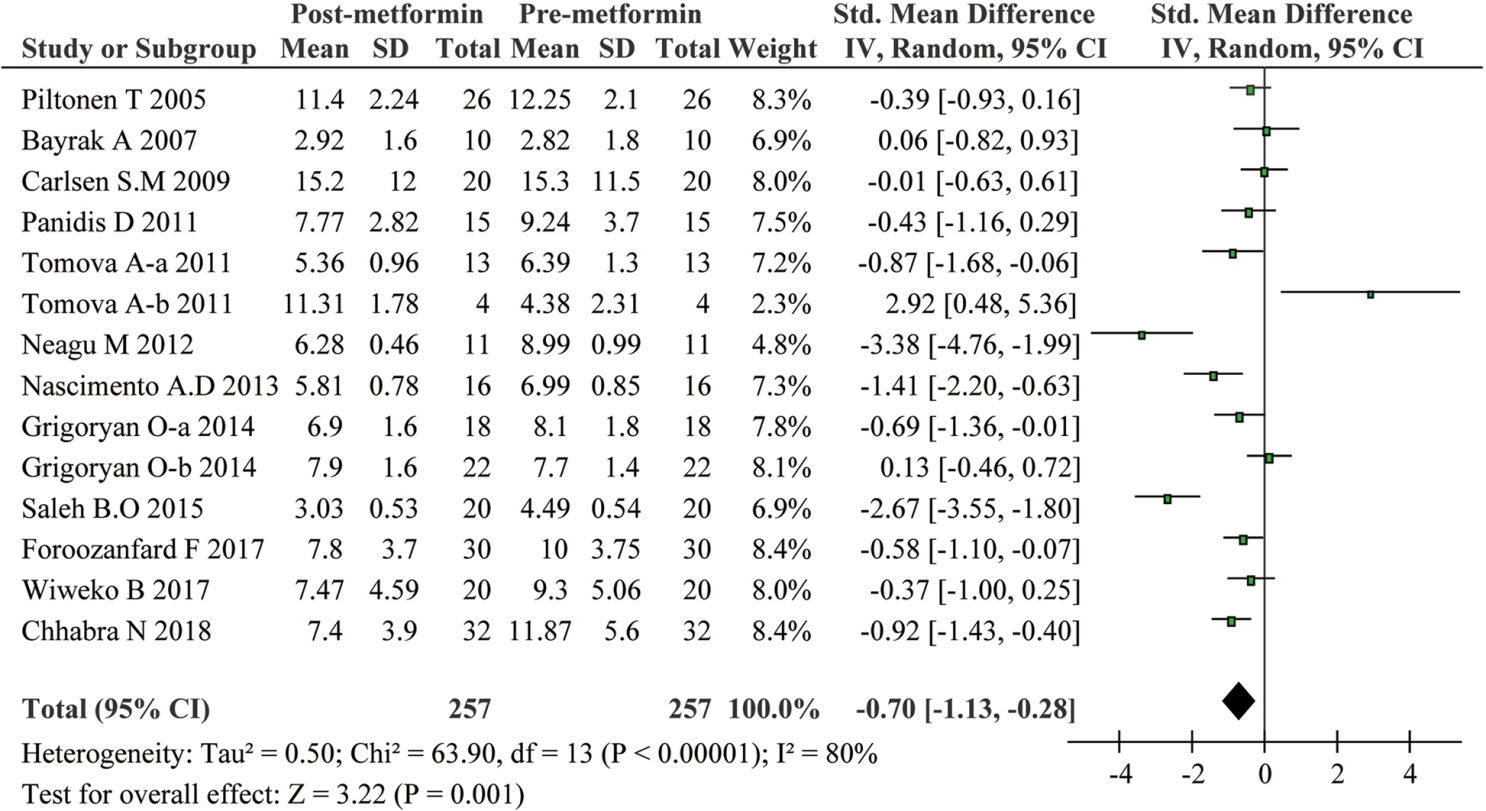
Meta-analysis of serum AMH levels in women with PCOS before and after metformin administration from 14 studies using a random-effect model. CI = confidence interval, AMH= anti-Müllerian hormone, PCOS = polycystic ovary syndrome, SMD = standard mean difference

As shown in Fig. 2, significant heterogeneity was found for the included studies (*I*^2^ = 80%, *P* < 0.00001). Thus, a random-effects model was applied to combine effect size. Our meta-analysis demonstrated a significant decrease in the serum AMH levels in patients with PCOS after metformin treatment (SMD -0.70, 95% CI -1.13 to -0.28, *P* = 0.001).

**Fig. 3:**
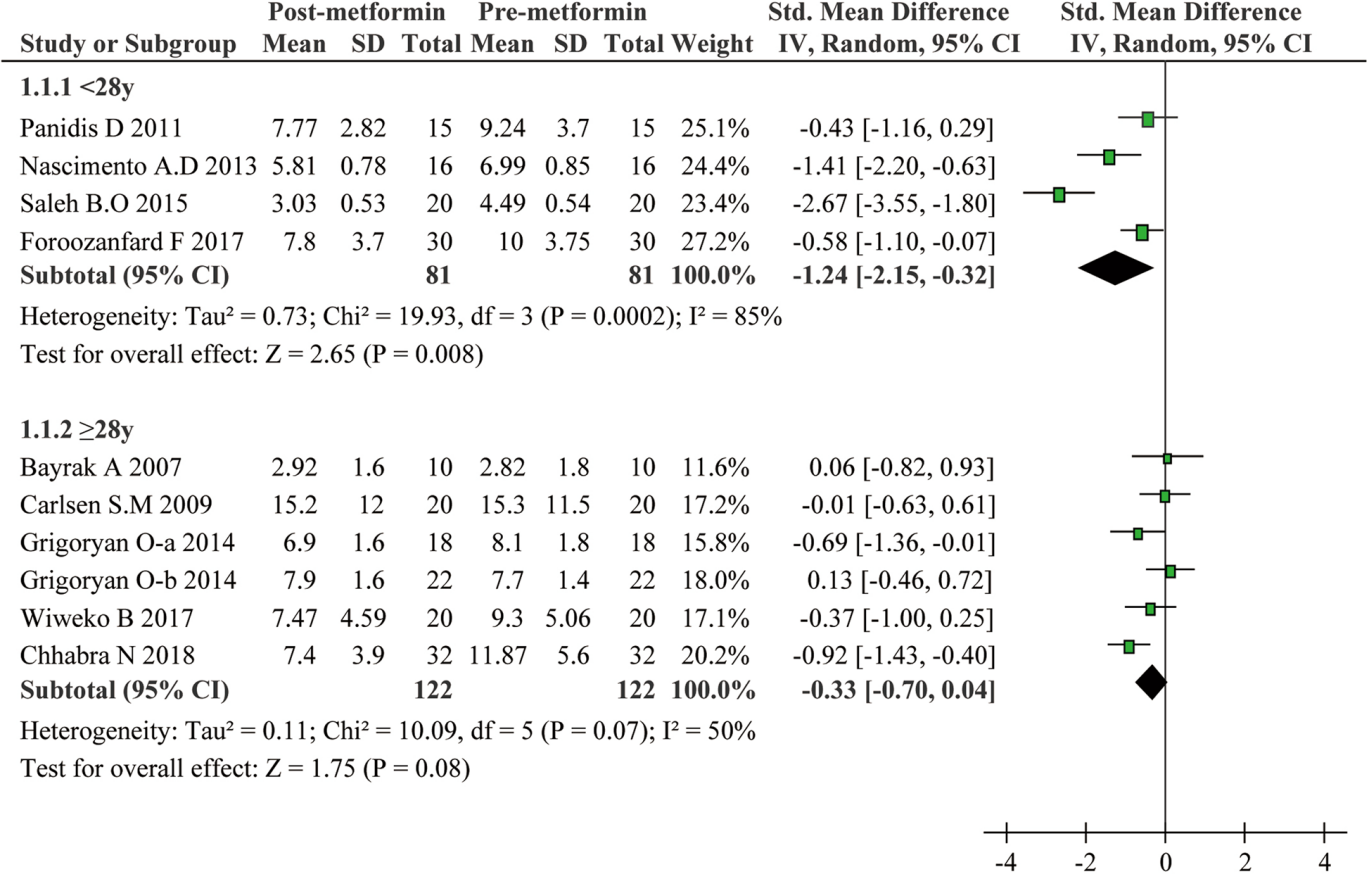
Forest plot for subgroup analysis stratified by age

**Fig. 4:**
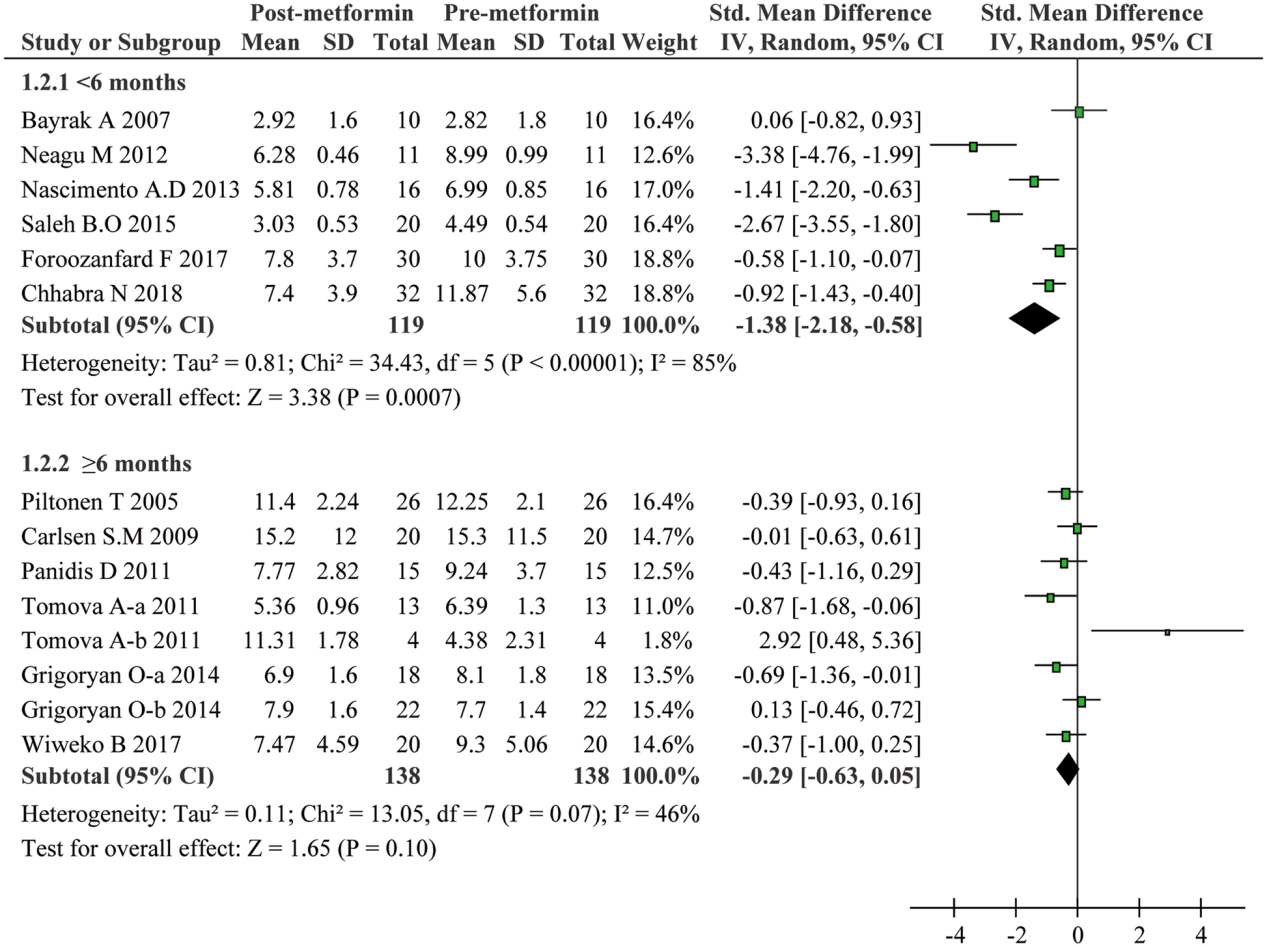
Forest plot for subgroup analysis stratified by treatment duration

**Fig. 5:**
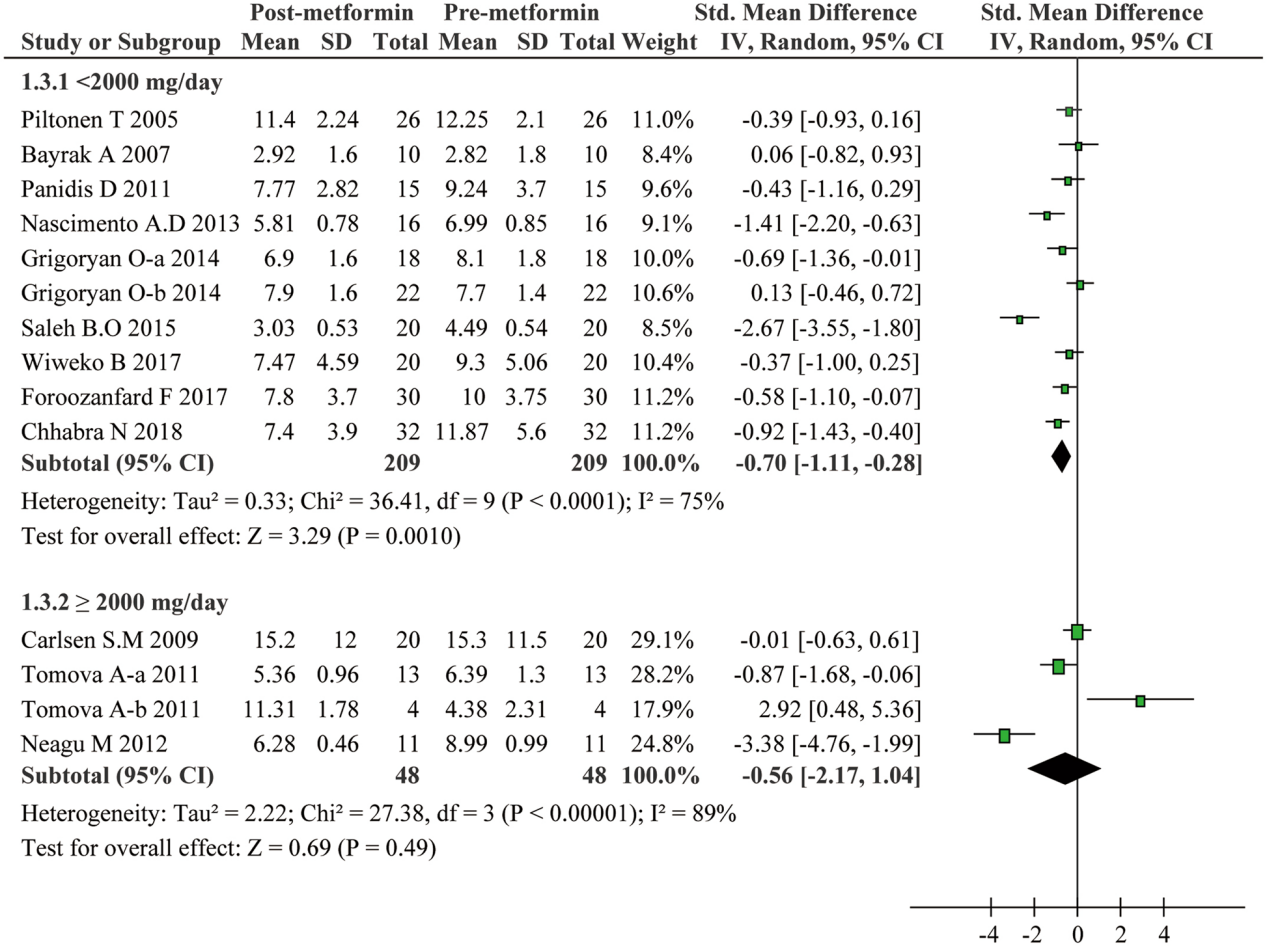
Forest plot for subgroup analysis stratified by treatment dose

**Fig. 6:**
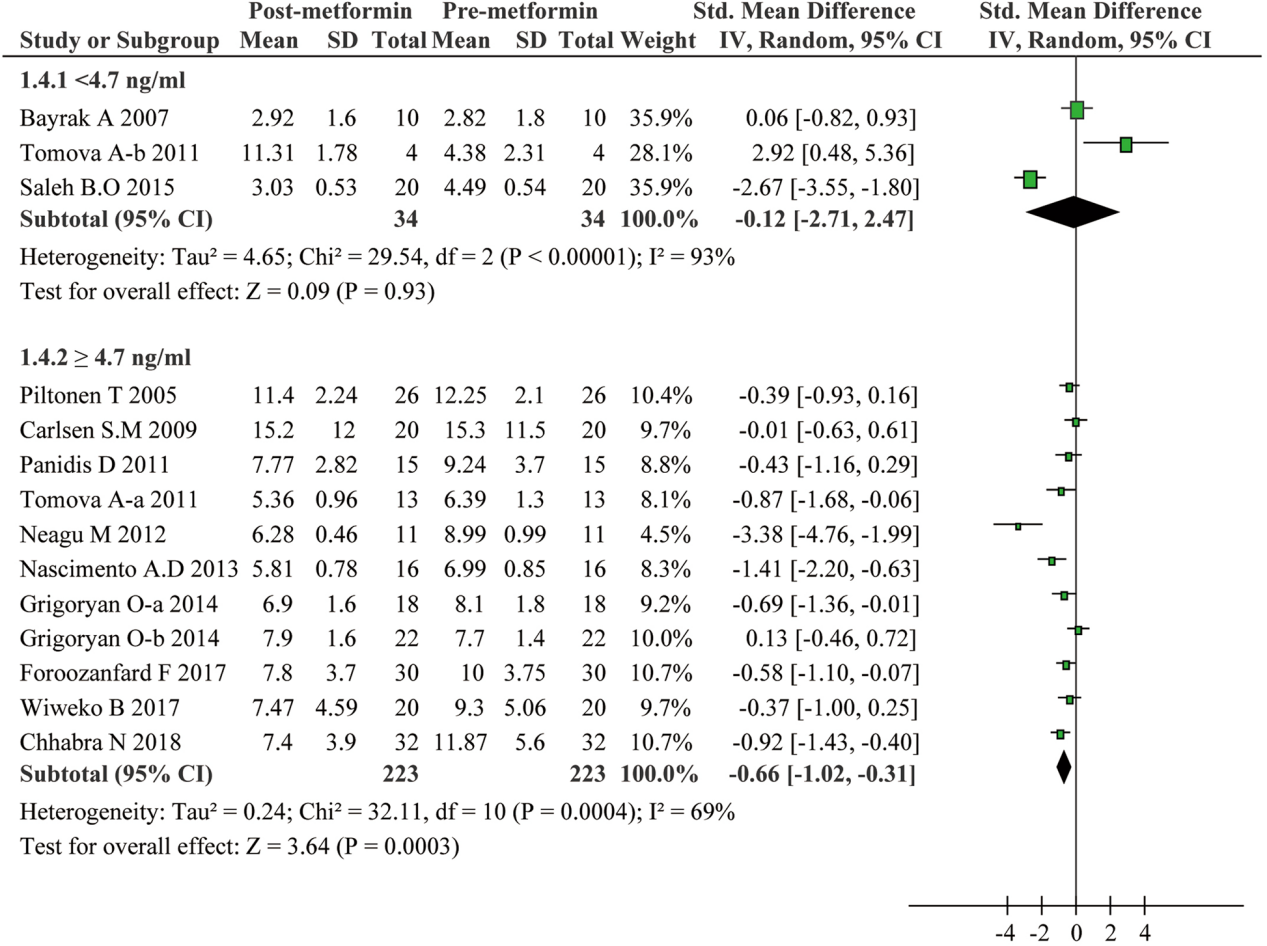
Forest plot for subgroup analysis stratified by the AMH levels at baseline

The included studies in the present meta-analysis displayed substantial heterogeneity regarding the effect of metformin on the serum AMH levels. Considering that treatment duration and dose of metformin as well as patients’ age and AMH levels at baseline could be critical factors affecting the therapeutic effect and cause extensive heterogeneity, we conducted subgroup analyses: Age was categorized as < 28 years and ≥ 28 years; dose of metformin as < 2000 mg/day and ≥ 2000 mg/day; treatment duration of metformin as < 6 months and ≥ 6 months, and the baseline of AMH as < 4.7 ng/ml and ≥ 4.7 ng/ml. These subgroup analyses led to interesting findings. On one hand, as presented in Fig. 3, metformin exhibited a strong inhibitory effect on AMH levels for PCOS patients with age less than 28 (SMD − 1.24, 95%CI − 2.15 to − 0.32, *P* = 0.008). On the other hand, metformin can hardly reduce the AMH levels in patients with age of 28 or more (SMD − 0.33, 95%CI − 0.70 to 0.04, *P* = 0.08). Additionally, serum AMH levels significantly slid down in PCOS patients with no more than 6 months metformin treatment (SMD − 1.38, 95%CI − 2.18 to − 0.58, *P* = 0.0007) (Fig. 4), or with no more than a dose of 2000 mg/day (SMD -0.70, 95% CI -1.11 to -0.28, *P* = 0.001) (Fig. 5). Unexpectedly, prolonged metformin treatment or higher daily dose of metformin failed to change AMH levels in PCOS patients. To determine whether the AMH levels at baseline could affect the outcomes of metformin treatment, subgroup analysis was performed based on the levels of AMH. We used 4.7 ng/ml as a cut-off value as suggested in previous study [22]. As shown in Fig. 6, metformin treatment led to a significant decrease of AMH levels in patients with AMH levels at baseline higher than 4.7 ng/ml. Conversely, this effect was not observed in patients with lower AMH levels. Nevertheless, there were still significant heterogeneity in these subgroup analyses, suggesting the high complexity of AMH regulation upon metformin treatment.

Pooled analysis using the random effects model demonstrated that metformin treatment significantly reduced AMH levels in PCOS patients (SMD -0.70, 95% CI -1.13 to -0.28, *P* = 0.001). The fixed-effects model analysis was also conducted and produced similar outcome (SMD -0.62, 95% CI -0.80 to -0.43, *P* < 0.00001, Supplementary Fig. 1). Sensitivity analysis was carried out and the result showed that deleting any of the included studies did not change the outcome of the existing pooled analyses (Supplementary Fig. 2, 3, 4, 5, 6, 7, 8, 9, 10, 11, 12, 13, 14, 15). Although the funnel plot was not perfectly symmetrical (Supplementary Fig. 16 A), the Begg’s test (*P* = 0.827) and Egger’s test (*P* = 0.370) revealed little evidence of publication bias (Supplementary Fig. 16 B ~ D).

## Discussion

Though metformin is not used as first-line agent for PCOS therapy, it exerts beneficial roles in ovulation induction [23] as well as improving insulin sensitivity [7]. The mechanisms by which metformin improves ovarian function remain not fully understood. A widely accepted theory is that metformin could improve reproductive function by relieving insulin resistance. But metformin was recommended for all PCOS patients despite of pre-treatment insulin sensitivity [24], which indicated an insulin-independent role of metformin per se. In 2005, Dr. Fleming and colleague reported the first study demonstrating an efficacy of metformin in reducing AMH levels in PCOS patients [25]. In Tomova’s study [16], patients who restored regular menstrual cycles after metformin treatment revealed 16.27% decrease of AMH levels, but AMH levels almost tripled instead of slid in those without satisfactory clinical response to metformin treatment. A number of clinical investigations related with AMH and metformin have been conducted but the outcomes were not consistent. All these studies were small-scale trials. Thus systematic review with meta-analysis is needed to combine these evidences. Results of the current meta-analysis showed that circulating AMH levels significantly reduced in PCOS patients after metformin therapy, suggesting an improvement of polycystic ovarian morphology. Yin and colleagues retrieved consistent result in their study [26]. Unlike their point of interest, subgroup analysis was conducted in the present study, which would arouse an interest to clarify the detail mechanism by which metformin regulates AMH levels in PCOS patients. As HOMA-IR values of pre- and post- treatment were not provided in the collected studies, we were not able to determine whether metformin suppressed AMH levels independent of insulin sensitivity alteration. Notably, a recent clinical investigation revealed similar AMH levels between insulin resistant PCOS patients and those with normal insulin sensitivity [27]. Plus the authors did not observe a correlation between AMH levels and insulin resistance index [27]. Thus, high AMH levels and insulin resistance might contribute to the pathogenesis of PCOS independently.

The subgroup analyses results showed that the inhibitory effects of metformin were largely compromised in subgroups of patients with age over 28, or treatment duration longer than 6 months, or treatment dose higher than 2000 mg/day, or the AMH levels at baseline were lower than 4.7 ng/ml. As age is the predominant fertility factor, it is not surprise that metformin treatment achieved a better outcome in younger patients. The outcomes of subgroup analyses based on treatment dose or duration, nevertheless, are unexpected. We speculate that PCOS patients prescribed with higher metformin dose or longer treatment might suffer from a more severe disease status such as severe insulin resistance. It’s unlikely that increasing the dose and/or an extension of treatment would reverse the effect of metformin on AMH levels. Thus, the AMH-lowering effect of metformin treatment may not be easily detected in these subjects. Nonetheless, we cannot exclude the possibility that high dose and/or long-term metformin treatment potentially lead to side effects which counteract the therapeutic effect in normalizing AMH levels. Based on these subgroup analyses, it should be safe to conclude that the severity of disease in PCOS patients could foretell the efficacy of metformin treatment. Additionally, it is worthy to note that metformin treatment has little effect in PCOS patients with AMH levels at baseline lower than 4.7 ng/ml. Since AMH levels at physiological range indicate ovarian reserve [2], this result indicates that metformin will not jeopardize ovarian function of PCOS patients with low AMH levels. The NIH criteria were sometimes employed for PCOS diagnosis and the patients in those studies exhibited low AMH levels [17]. The etiology of PCOS as well as the regulation of AMH in these patients could be distinct. But for patients with polycystic ovarian morphology, AMH may be a good prognostic marker of therapeutic efficiency in the treatment of infertility and polycystic ovary syndrome. As high AMH levels were reported to associate with compromised response to progesterone and clomiphene citrate treatment [28, 29], metformin might hold an advantageous position in PCOS patients with high AMH levels and could be used in combination with other agents as first-line therapy for ovulation induction. The results of this meta-analysis could provide valuable evidence in clinical practice, especially when AMH is used as prognostic biomarker for PCOS patients in metformin treatment.

For the enrolled studies, we also noticed that methods applied for determining AMH levels were not alike. As a promising biomarker of ovarian function, a global standard for AMH measurement is in need urgently to maximize its utility in PCOS diagnosis and therapy.

In conclusion, results of the current meta-analysis suggested that metformin treatment could decrease serum AMH levels in patients diagnosed with PCOS. Patients’ ages and AMH levels, as well as dose and duration of metformin treatment, should be taken into consideration in clinical practice. As the sample sizes of the collected studies in this meta-analysis are small, well designed large-scale RCT studies in PCOS patients are still necessary to affirm these findings.

## Supplementary Information

**Additional file 1. **

**Additonal file 2:** **Supplementary Table 2.** Quality assessment of studies pooled in the meta-analysis based on the Newcastle-Ottawa Scale judgment.

**Additional file 3:** **Supplementary Figure 1.** Meta-analysis of serum AMH levels in women with PCOS before and after metformin administration from 14 studies using a fixed-effect model.

**Additional file 4:** **Supplementary Figure 2**-**15.** Sensitivity analysing of serum AMH levels in women with PCOS before and after metformin administration using a random-effect model by excluding the studies one by one.

**Additional file 5:** **Supplementary Figure 16.** Publication bias analysis.

**Additional file 6. **
