# Supplementary material for: The effects of metformin on anti-Müllerian hormone levels in patients with polycystic ovary syndrome: a systematic review and meta-analysis
Source: J Ovarian Res. 2023 Jun 28;16:123. doi: 10.1186/s13048-023-01195-1 (PMC10303859; doi:10.1186/s13048-023-01195-1)
Supplement: Supplementary file 2 — Additonal file 2: Supplementary Table 2. Quality assessment of studies pooled in the meta-analysis based on the Newcastle-Ottawa Scale judgment. [file 13048_2023_1195_MOESM2_ESM.docx]

| **study** | **selection** | | | | **comparability** | **outcome** | | |  |
| --- | --- | --- | --- | --- | --- | --- | --- | --- | --- |
|  | **Representativeness of the exposed cohort** | **Selection of the non exposed cohort** | **Ascertainment of exposure** | **Demonstration that outcome of interest was not present at start of study** | **Comparability of cohorts on the basis of the design or analysis** | **Assessment of outcome** | **Was follow-up long enough for outcomes to occur** | **Adequacy of follow up of cohorts** | **Quality score** |
| Piltonen 2005 | * | * | * | * | * | * |  |  | ****** |
| Bayrak 2007 | * | * | * | * | * | * |  |  | ****** |
| Carlsen 2009 | * | * | * | * | * | * |  |  | ****** |
| Panidis 2011 | * | * | * | * | * | * |  |  | ****** |
| Tomova 2011 | * | * | * | * | * | * |  | * | ******* |
| Neagu 2012 | * | * | * | * | * | * |  |  | ****** |
| Nascimento 2013 | * | * | * | * | * | * |  |  | ****** |
| Grigoryan 2014 | * | * | * | * | * | * |  |  | ****** |
| Saleh 2015 | * | * | * | * | * | * | * |  | ******* |
| Wiweko 2017 | * | * | * | * | * | * |  |  | ****** |
| Foroozanfard 2017 | * | * | * | * | * | * |  |  | ****** |
| Chhabra 2018 | * | * | * | * | * | * |  |  | ****** |

**Supplementary Table 2. Quality assessment of studies pooled in the meta-analysis based on the Newcastle-Ottawa Scale judgment.**
