# Supplementary figures and images for: The effects of metformin on anti-Müllerian hormone levels in patients with polycystic ovary syndrome: a systematic review and meta-analysis
Source: J Ovarian Res. 2023 Jun 28;16:123. doi: 10.1186/s13048-023-01195-1 (PMC10303859; doi:10.1186/s13048-023-01195-1)

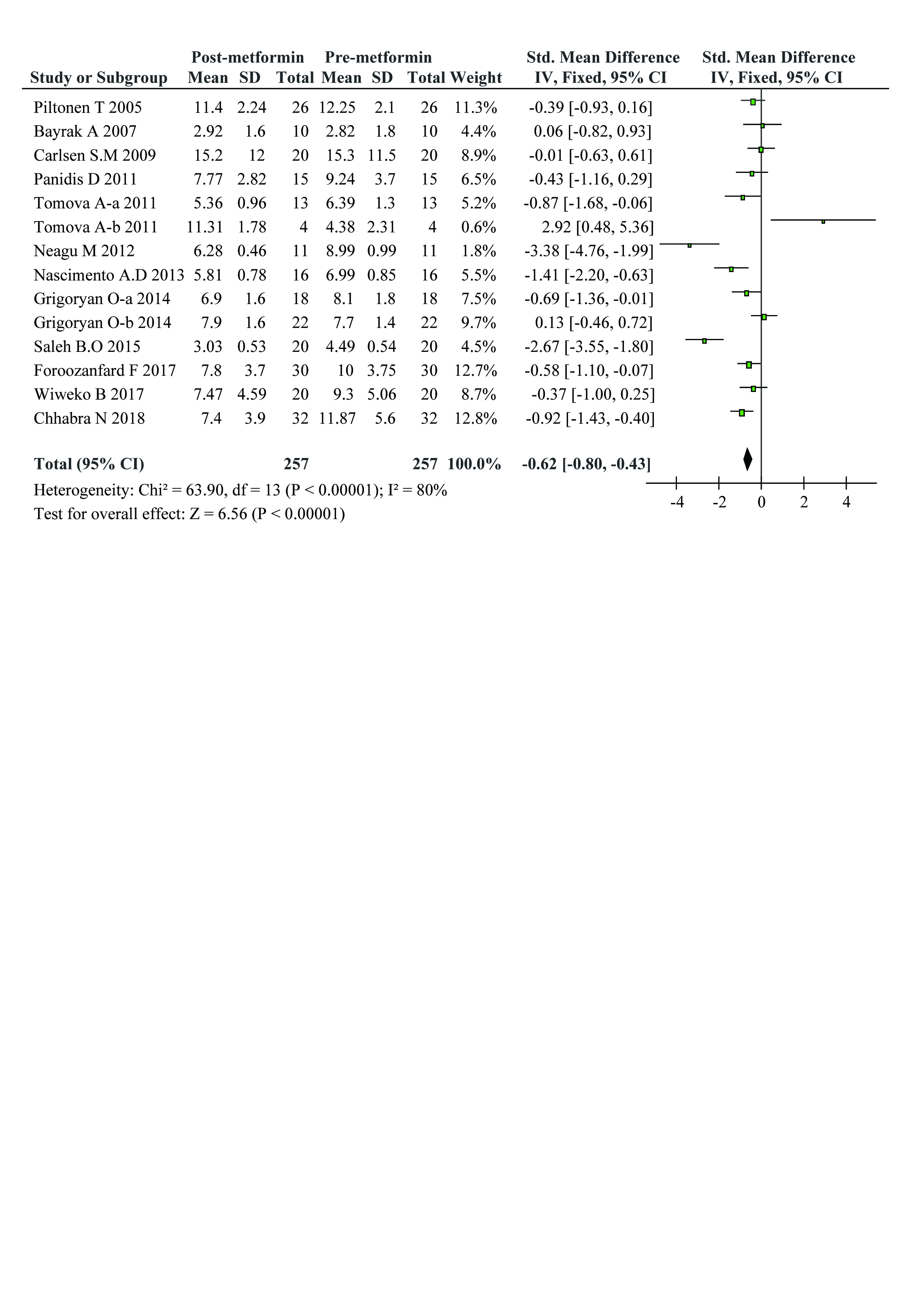

Supplement: Supplementary file 3 — Additional file 3: Supplementary Figure 1. Meta-analysis of serum AMH levels in women with PCOS before and after metformin administration from 14 studies using a fixed-effect model. [file 13048_2023_1195_MOESM3_ESM.jpg]

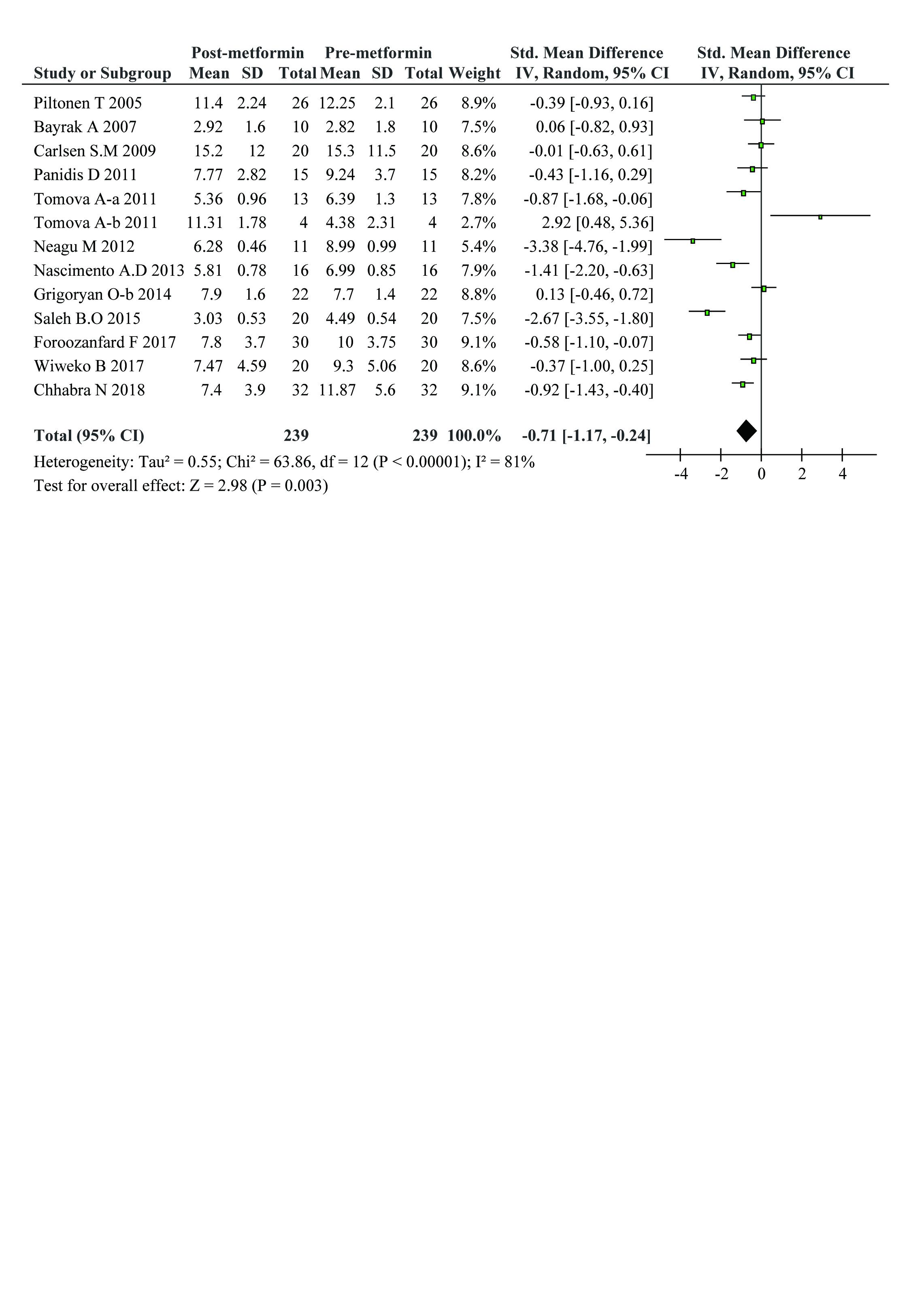

Supplement: Supplementary file 4 — Additional file 4: Supplementary Figure 2-15. Sensitivity analysing of serum AMH levels in women with PCOS before and after metformin administration using a random-effect model by excluding the studies one by one. [file 13048_2023_1195_MOESM4_ESM.zip › Supplementary Figure 10-20230515.jpg]

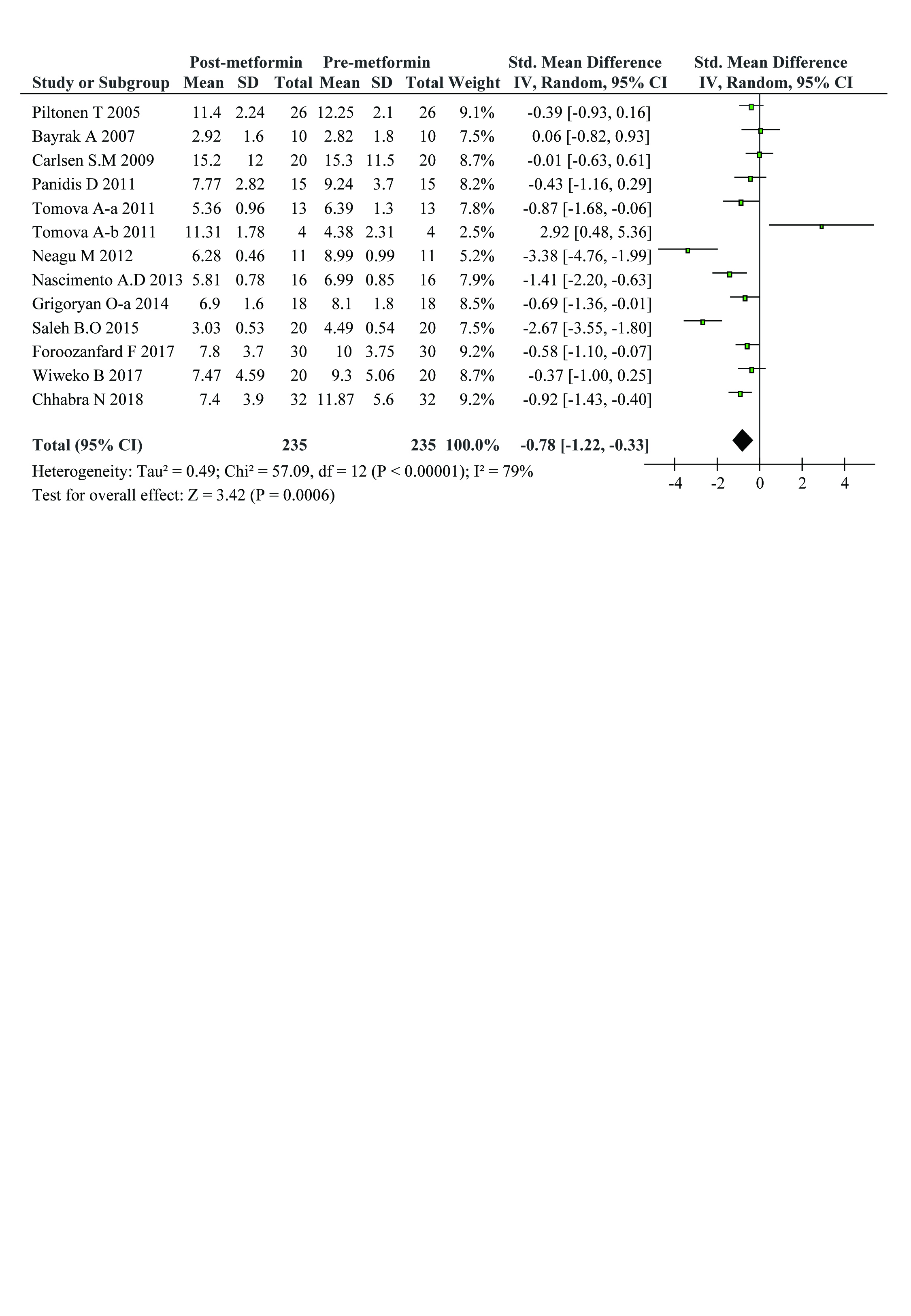

Supplement: Supplementary file 4 — Additional file 4: Supplementary Figure 2-15. Sensitivity analysing of serum AMH levels in women with PCOS before and after metformin administration using a random-effect model by excluding the studies one by one. [file 13048_2023_1195_MOESM4_ESM.zip › Supplementary Figure 11-20230515.jpg]

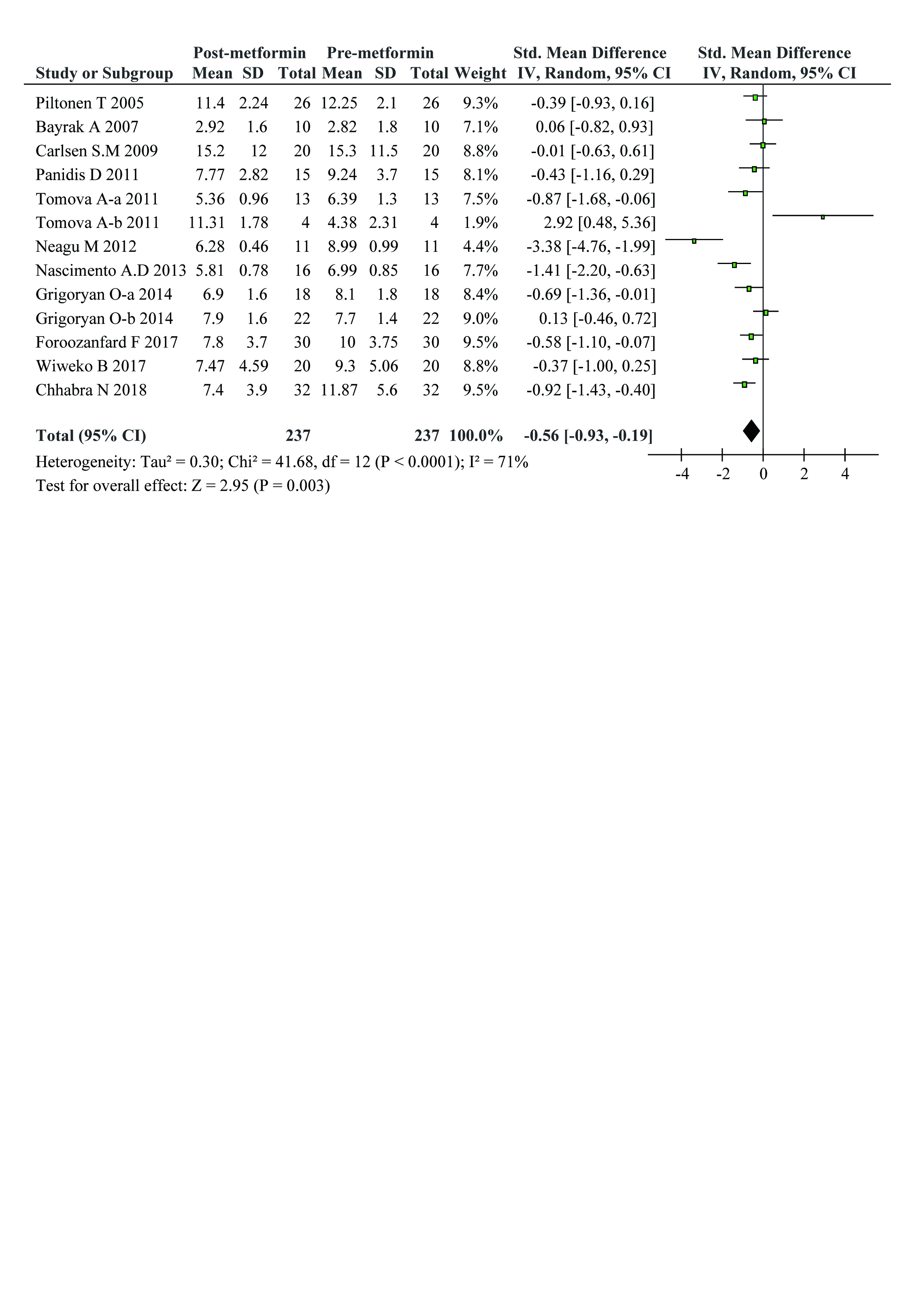

Supplement: Supplementary file 4 — Additional file 4: Supplementary Figure 2-15. Sensitivity analysing of serum AMH levels in women with PCOS before and after metformin administration using a random-effect model by excluding the studies one by one. [file 13048_2023_1195_MOESM4_ESM.zip › Supplementary Figure 12-20230515.jpg]

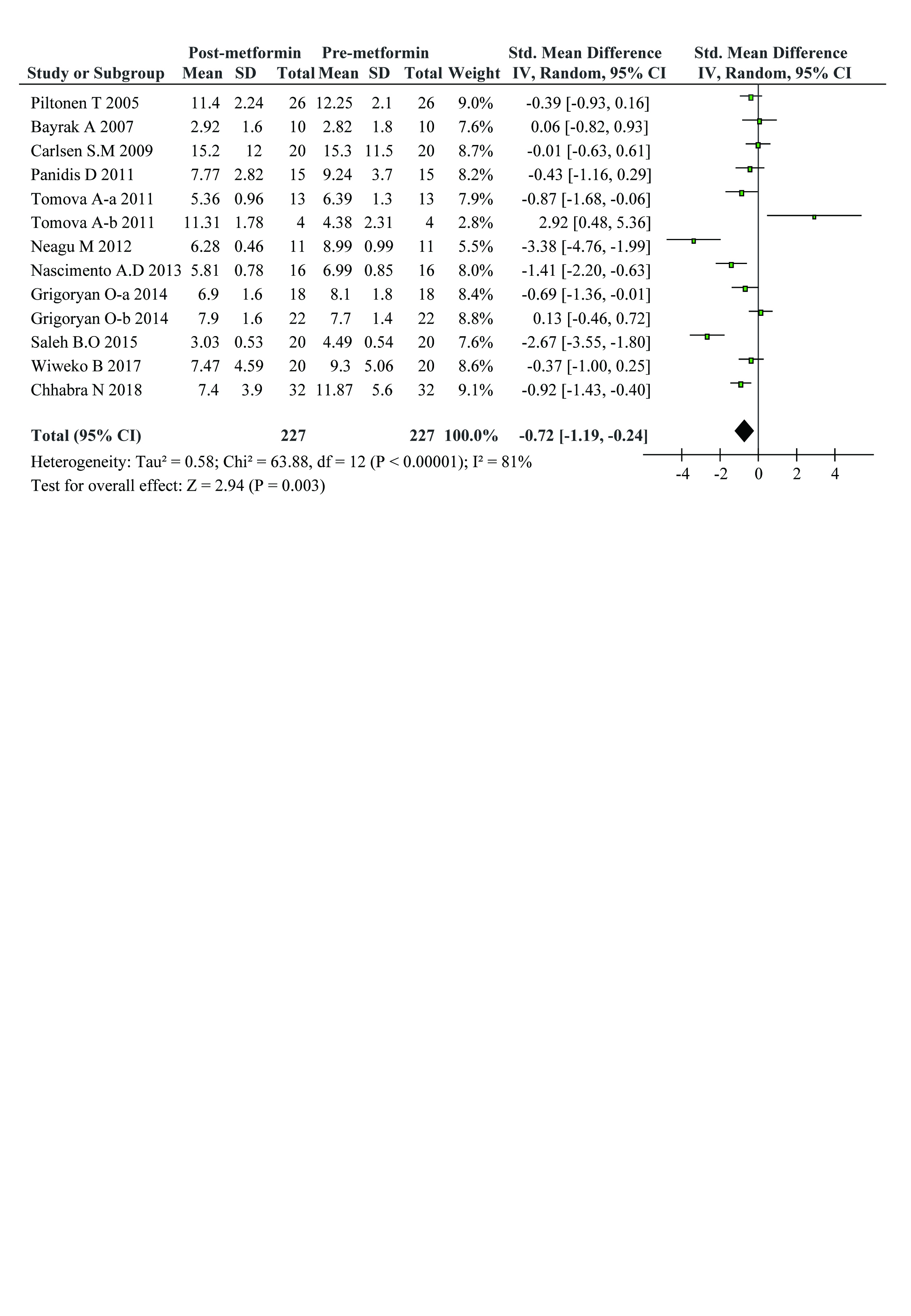

Supplement: Supplementary file 4 — Additional file 4: Supplementary Figure 2-15. Sensitivity analysing of serum AMH levels in women with PCOS before and after metformin administration using a random-effect model by excluding the studies one by one. [file 13048_2023_1195_MOESM4_ESM.zip › Supplementary Figure 13-20230515.jpg]

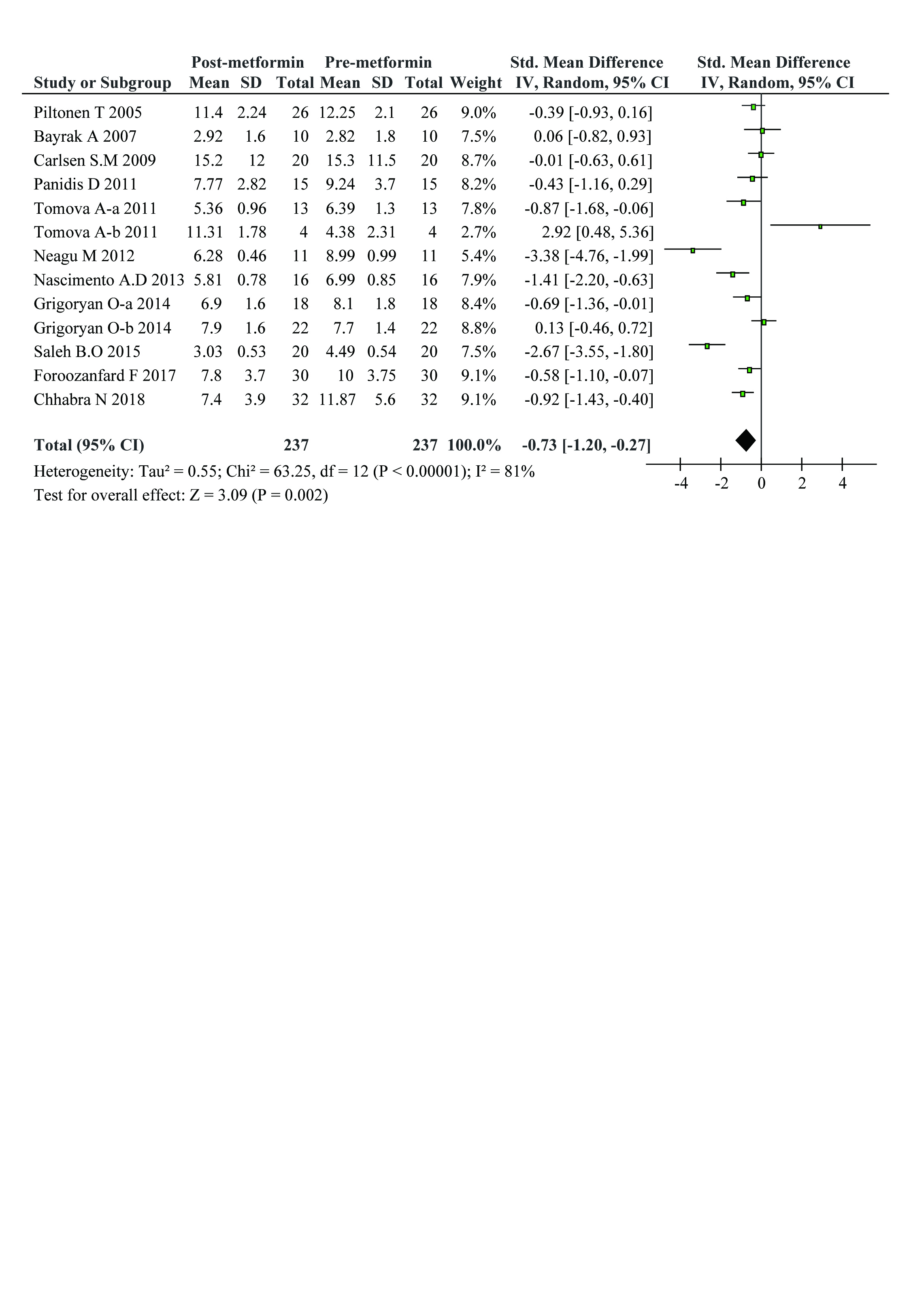

Supplement: Supplementary file 4 — Additional file 4: Supplementary Figure 2-15. Sensitivity analysing of serum AMH levels in women with PCOS before and after metformin administration using a random-effect model by excluding the studies one by one. [file 13048_2023_1195_MOESM4_ESM.zip › Supplementary Figure 14-20230515.jpg]

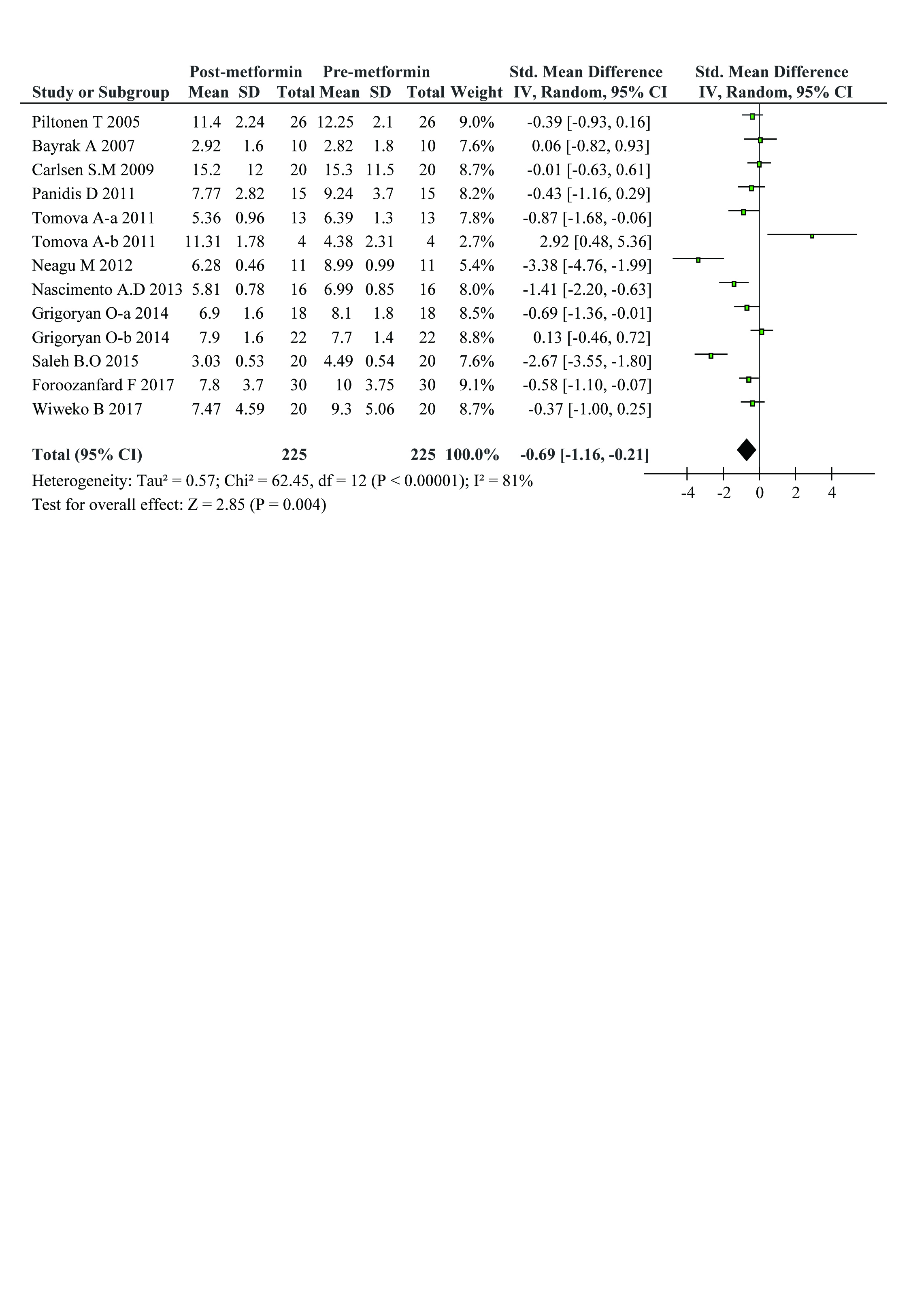

Supplement: Supplementary file 4 — Additional file 4: Supplementary Figure 2-15. Sensitivity analysing of serum AMH levels in women with PCOS before and after metformin administration using a random-effect model by excluding the studies one by one. [file 13048_2023_1195_MOESM4_ESM.zip › Supplementary Figure 15-20230515.jpg]

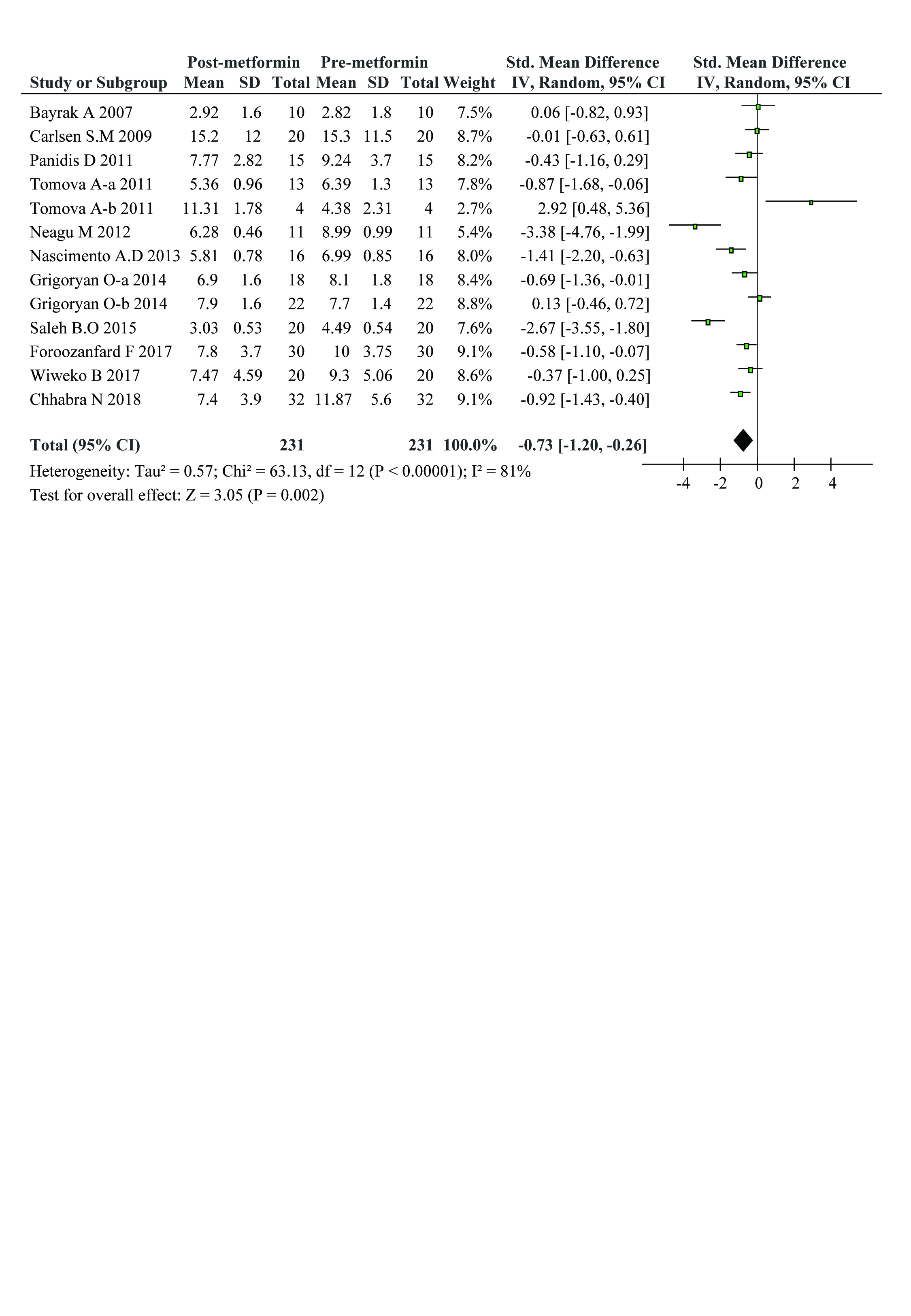

Supplement: Supplementary file 4 — Additional file 4: Supplementary Figure 2-15. Sensitivity analysing of serum AMH levels in women with PCOS before and after metformin administration using a random-effect model by excluding the studies one by one. [file 13048_2023_1195_MOESM4_ESM.zip › Supplementary Figure 2-20230515.jpg]

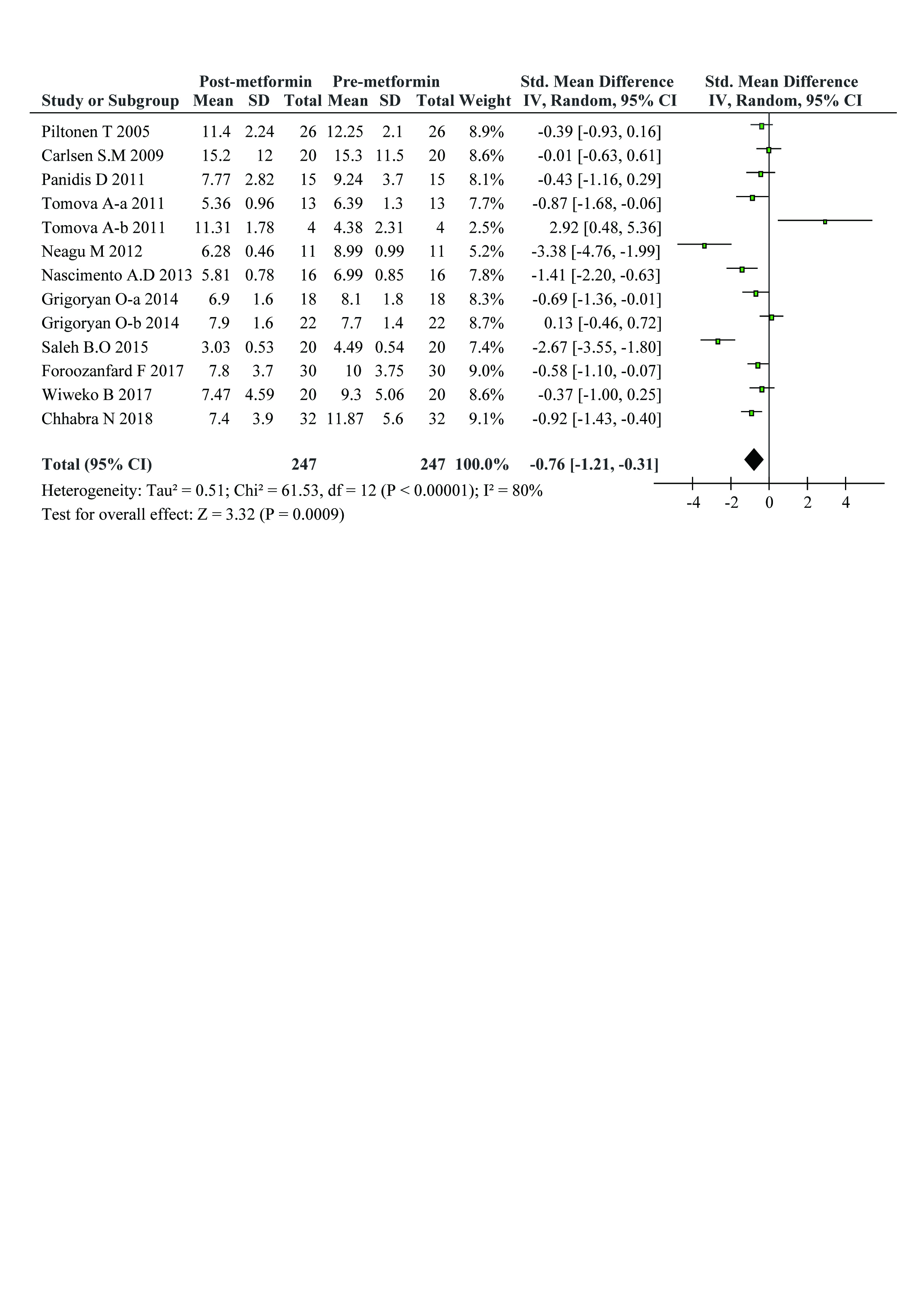

Supplement: Supplementary file 4 — Additional file 4: Supplementary Figure 2-15. Sensitivity analysing of serum AMH levels in women with PCOS before and after metformin administration using a random-effect model by excluding the studies one by one. [file 13048_2023_1195_MOESM4_ESM.zip › Supplementary Figure 3-20230515.jpg]

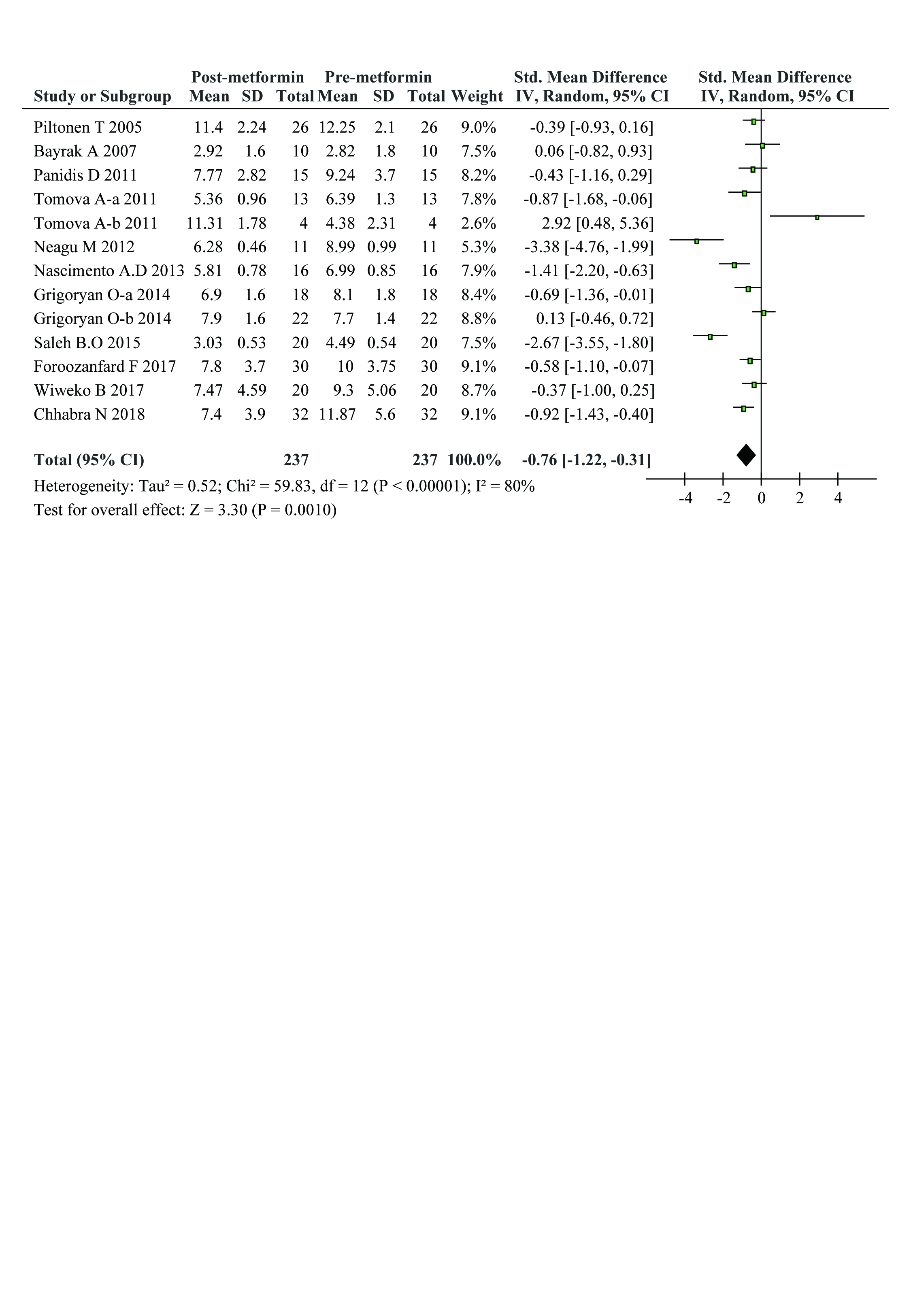

Supplement: Supplementary file 4 — Additional file 4: Supplementary Figure 2-15. Sensitivity analysing of serum AMH levels in women with PCOS before and after metformin administration using a random-effect model by excluding the studies one by one. [file 13048_2023_1195_MOESM4_ESM.zip › Supplementary Figure 4-20230515.jpg]

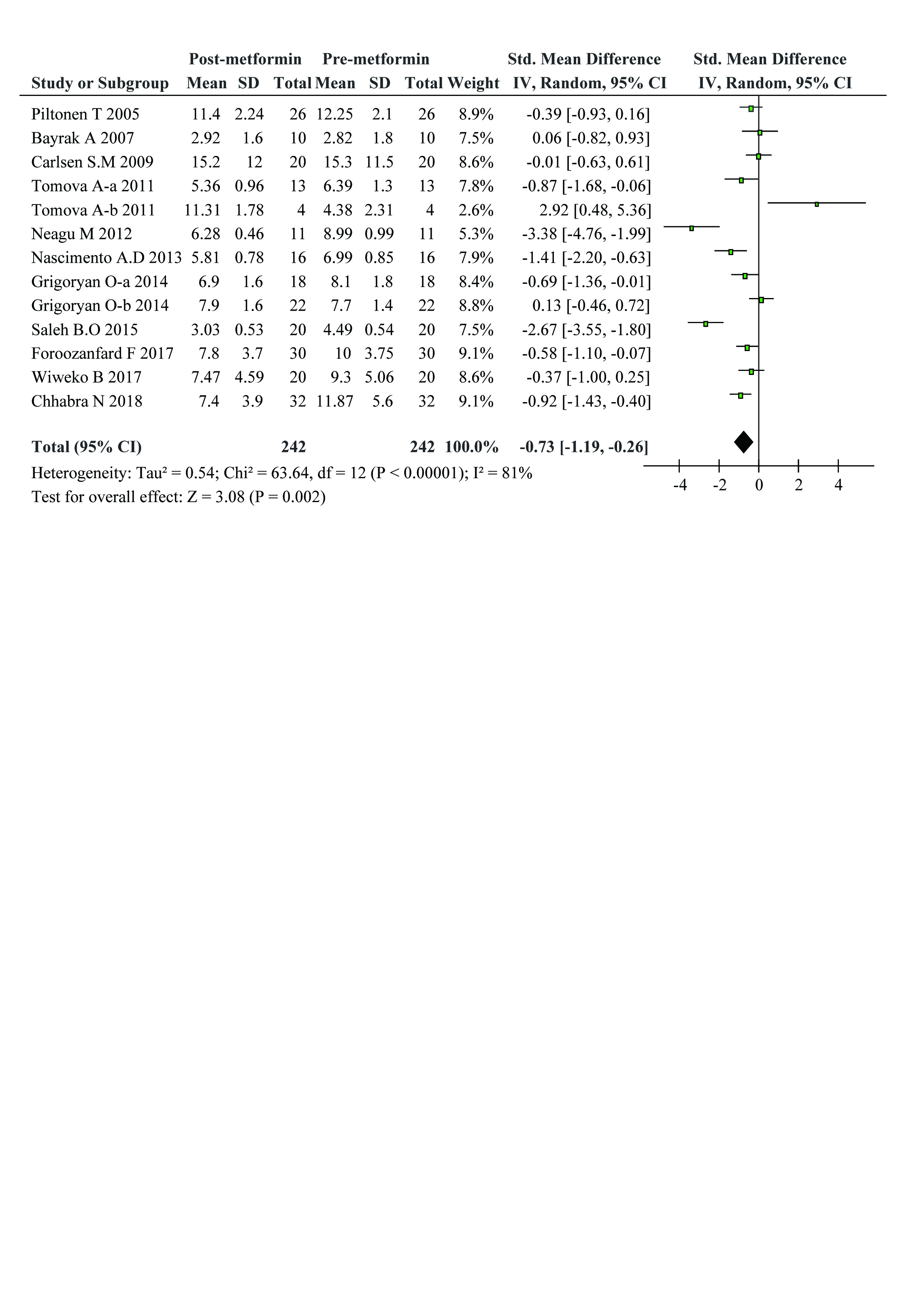

Supplement: Supplementary file 4 — Additional file 4: Supplementary Figure 2-15. Sensitivity analysing of serum AMH levels in women with PCOS before and after metformin administration using a random-effect model by excluding the studies one by one. [file 13048_2023_1195_MOESM4_ESM.zip › Supplementary Figure 5-20230515.jpg]

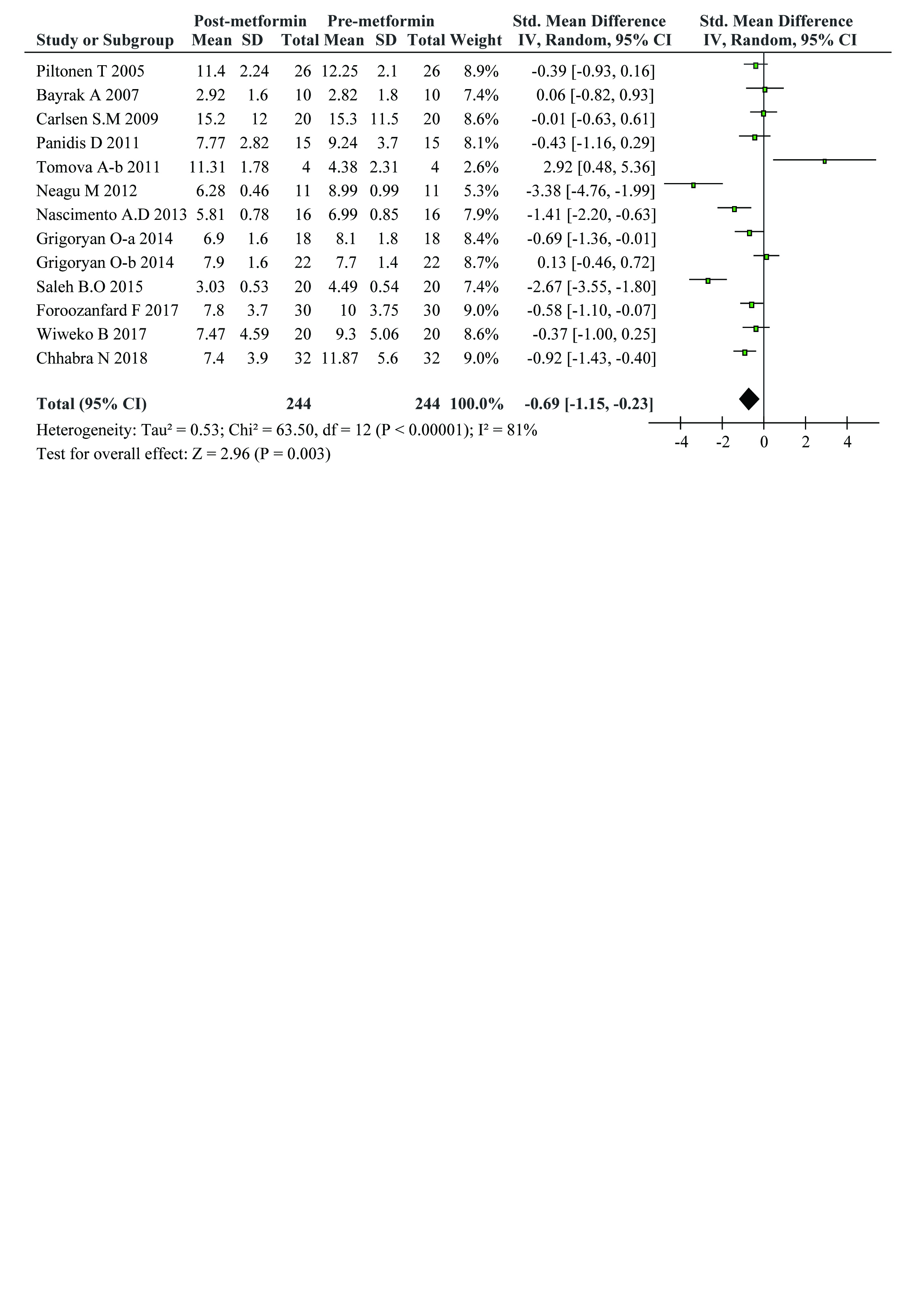

Supplement: Supplementary file 4 — Additional file 4: Supplementary Figure 2-15. Sensitivity analysing of serum AMH levels in women with PCOS before and after metformin administration using a random-effect model by excluding the studies one by one. [file 13048_2023_1195_MOESM4_ESM.zip › Supplementary Figure 6-20230515.jpg]

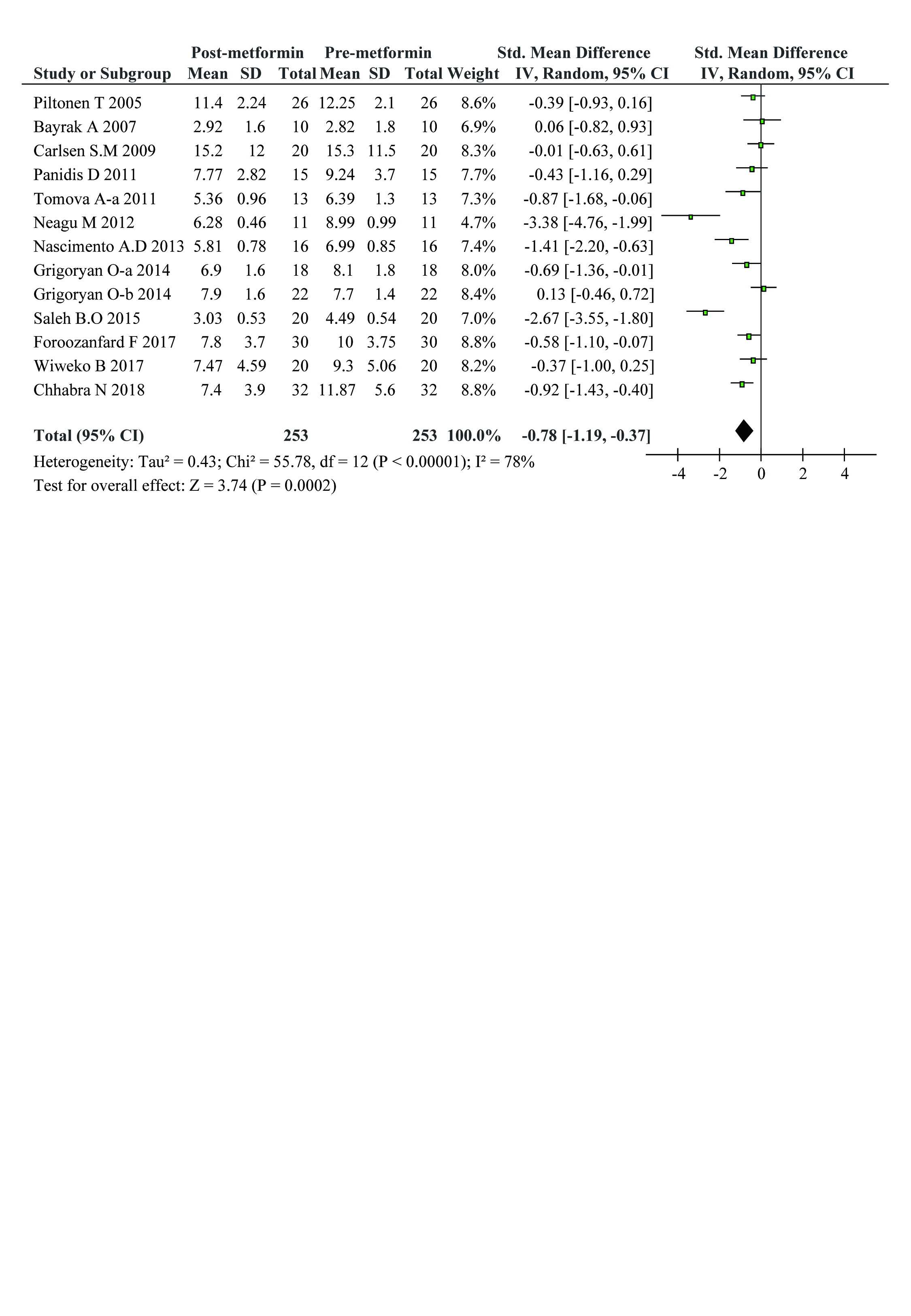

Supplement: Supplementary file 4 — Additional file 4: Supplementary Figure 2-15. Sensitivity analysing of serum AMH levels in women with PCOS before and after metformin administration using a random-effect model by excluding the studies one by one. [file 13048_2023_1195_MOESM4_ESM.zip › Supplementary Figure 7-20230515.jpg]

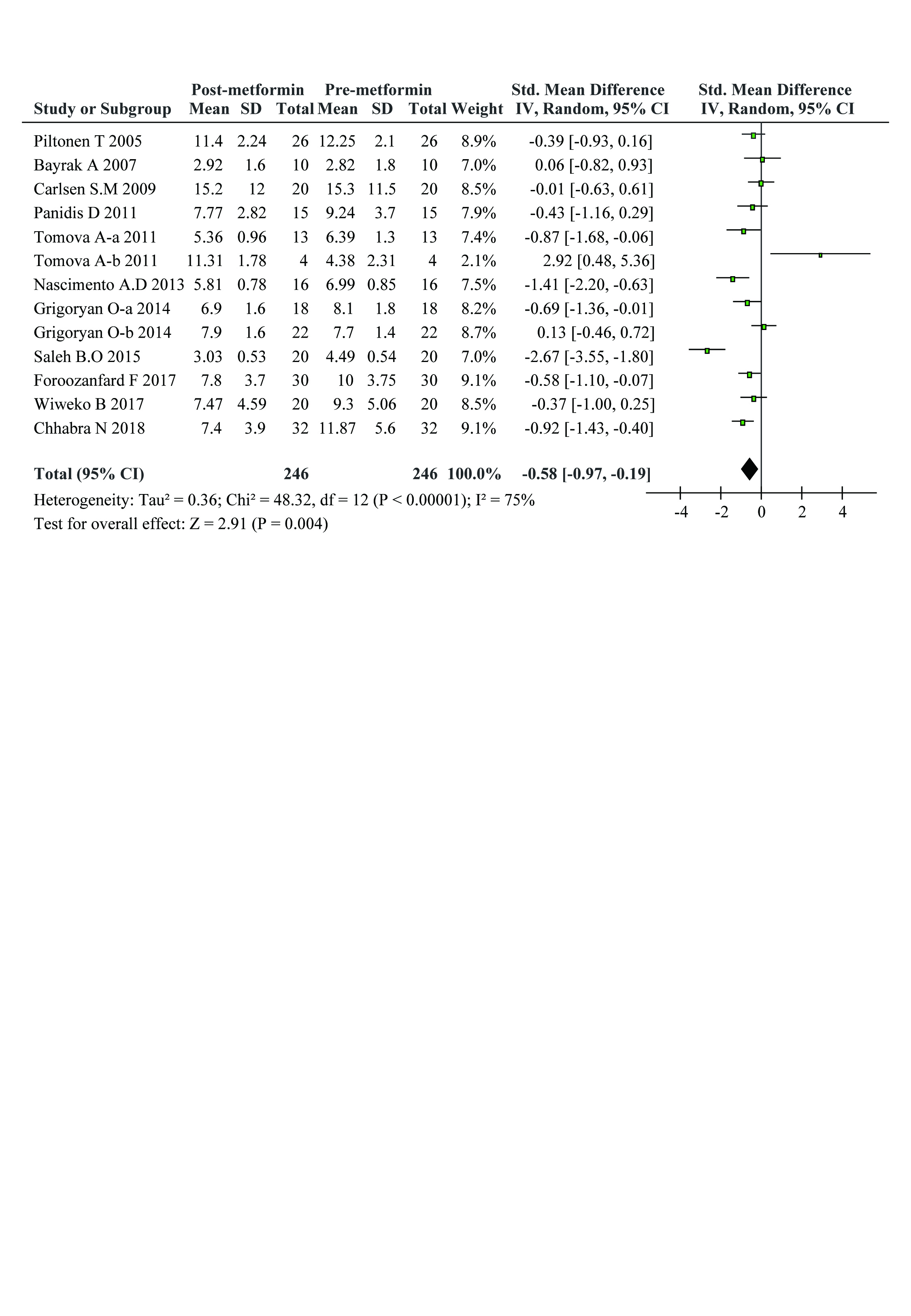

Supplement: Supplementary file 4 — Additional file 4: Supplementary Figure 2-15. Sensitivity analysing of serum AMH levels in women with PCOS before and after metformin administration using a random-effect model by excluding the studies one by one. [file 13048_2023_1195_MOESM4_ESM.zip › Supplementary Figure 8-20230515.jpg]

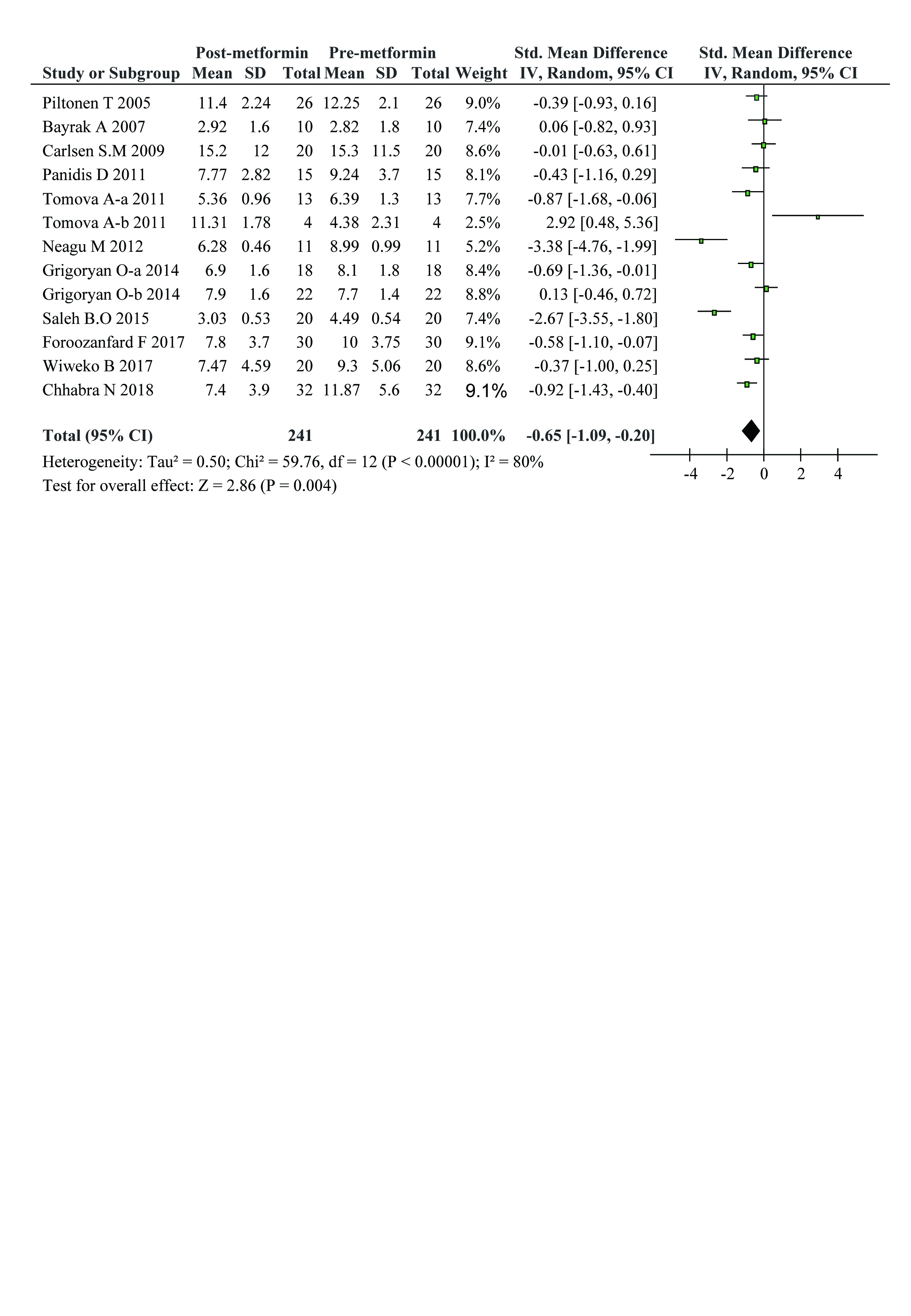

Supplement: Supplementary file 4 — Additional file 4: Supplementary Figure 2-15. Sensitivity analysing of serum AMH levels in women with PCOS before and after metformin administration using a random-effect model by excluding the studies one by one. [file 13048_2023_1195_MOESM4_ESM.zip › Supplementary Figure 9-20230515.jpg]

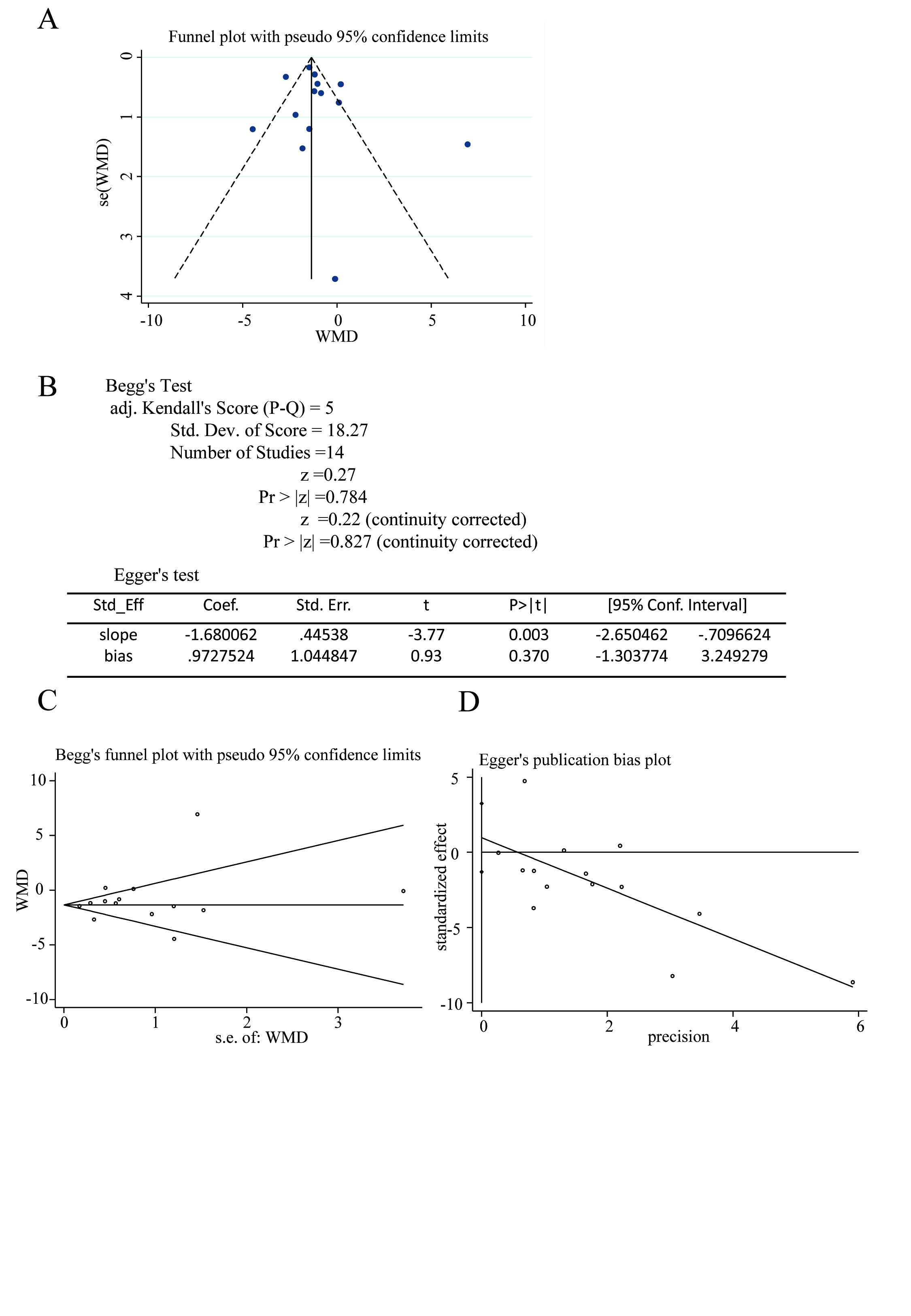

Supplement: Supplementary file 5 — Additional file 5: Supplementary Figure 16. Publication bias analysis. [file 13048_2023_1195_MOESM5_ESM.jpg]
