# Supplementary material for: The effects of metformin on anti-Müllerian hormone levels in patients with polycystic ovary syndrome: a systematic review and meta-analysis
Source: J Ovarian Res. 2023 Jun 28;16:123. doi: 10.1186/s13048-023-01195-1 (PMC10303859; doi:10.1186/s13048-023-01195-1)
Supplement: Supplementary file 6 — Additional file 6. [file 13048_2023_1195_MOESM6_ESM.docx]

Supplementary Methods

We searched the databases of PubMed, Embase, and Web of Science for some relevant articles inception to October 1, 2019 (updated on February 5, 2023) based on the search terms((anti-Müllerian hor,mone or AMH or Mullerian inhibiting substance or MIS) AND (metformin) AND (PCOS *or* polycystic ovary syndrome
